# Divergent TIR signaling domains in TLR7 and TLR9 control opposing effects on systemic autoimmunity

**DOI:** 10.1172/JCI189566

**Published:** 2025-08-12

**Authors:** Claire Leibler, Kayla B. Thomas, Coralie Josensi, Russell C. Levack, Shuchi Smita, Shinu John, Daniel J. Wikenheiser, Sheldon Bastacky, Sebastien Gingras, Kevin M. Nickerson, Mark J. Shlomchik

**Affiliations:** 1 Department of Immunology, University of Pittsburgh School of Medicine, Pittsburgh, Pennsylvania, USA.; 2 Univ. Bordeaux, CNRS, ImmunoConcEpT, and; 3 CHU de Bordeaux, Department of Immunology, UMR 5164, F-33000 Bordeaux, France.; 4 Department of Laboratory Medicine, Yale University School of Medicine, New Haven, Connecticut, USA.; 5 Department of Pathology, University of Pittsburgh School of Medicine, Pittsburgh, Pennsylvania, USA.

**Keywords:** Autoimmunity, Immunology, Innate immunity, Lupus

## Abstract

Toll like receptor (TLR) 7 and 9, endosomal sensors for RNA and DNA, are key mediators of autoreactivity. Although generally considered homologous, they paradoxically have opposing effects on lupus: TLR7 exacerbates the disease while TLR9 protects from it How they mediate opposing effects in autoimmunity remains undetermined. We hypothesized that differences in signaling qualities of the Toll-Interleukin 1 Receptor (TIR) domains of TLR7 and TLR9 could be responsible for their opposing effects. To test this, we introduced the TIR domain of TLR9 into the endogenous *Tlr7* locus and the TLR7 TIR domain into the endogenous *Tlr9* locus of mice, creating chimeric molecules termed TLR779 and TLR997. Lupus-prone MRL/lpr mice carrying *Tlr^779^* had greatly ameliorated disease, while MRL/lpr mice carrying *Tlr^997^* had markedly exacerbated disease compared with respective *Tlr^WT^* mice. These experiments establish that TLR7 and TLR9 TIR domains have divergent properties and control disease quality, thus explaining the longstanding “TLR paradox”.

## Abstract 2

Using germline domain swapping, we show that the Toll-IL1 Receptor (TIR) domains of TLR7 and TLR9 encode their divergent proinflammatory and regulatory properties in lupus

## Introduction

In systemic lupus erythematosus (SLE, lupus), endosomal toll like receptors (TLRs), TLR7 and TLR9, that sense (self) RNA and DNA, respectively, by signaling via activation of MyD88, initiate the loss of self tolerance and control disease severity (1). GWAS studies have mapped more than half of the SLE-associated loci to TLR signaling pathways (2). Notably, gain-of-function TLR7 mutations promote lupus in mice (3) and humans (4). While deletion of TLR7 ameliorates lupus in animal models, TLR9 deficiency exacerbates disease, revealing an unexpected protective role of TLR9 (5, 6). Further, both TLR7 and TLR9 must be specifically expressed in B cells to mediate these effects (7, 8). The opposing effects of TLR7 and TLR9 on lupus disease has been termed the “TLR Paradox”. Recently, by mutating either the ligand-binding or MyD88-binding region of the Toll-IL1R (TIR) domain of TLR9, we showed that the protective effects of TLR9 were likely comprised of both a ligand-independent component as well as a ligand-dependent but MyD88-signaling–independent component. These studies also showed that intact TLR9 additionally possessed a proinflammatory MyD88-dependent activity (9).

There are several nonexclusive explanations of the TLR Paradox. In B cells, TLR7 and TLR9 are localized to different endosomes (9), which could lead to different downstream signaling (10–12), albeit that one type of signaling must be regulatory in nature. Localization of TLRs is thought to be enforced by the transmembrane and juxtamembrane domains (13). Another possible difference is that differential modes of ligand recognition and/or duration of ligand binding drive TLR7 and TLR9 functional differences; these may map to the ligand-binding ectodomain. Finally, the TIR domains of TLR7 and TLR9 — which share only 42% sequence identity — could have fundamentally different properties that could encode meaningfully different downstream signaling and, thus, disease outcomes in the context of lupus. This would be TIR-domain encoded and independent of other differences such as localization or ligand binding. If the negative regulatory signaling pathway that we previously implicated is unique to TLR9, it could explain, in part, why TLR9 and TLR7 are functionally different.

To test the hypothesis that differential regulatory and proinflammatory functions of TLR7 and TLR9 are encoded by TIR domains, we created 2 chimeric TLRs in which the TIR-signaling domains were swapped: TLR779, which binds RNA and localizes in TLR7 endosomes but signals like TLR9; and TLR997, which binds DNA, localizes in TLR9 endosomes, but signals like TLR7. We inserted the chimeric TLRs into the endogenous locus of TLR7 and TLR9, respectively, in lupus-prone MRL/lpr mice and then studied signaling, TLR ligand-inducible gene expression, TLR localization, and, finally, the effect of the TIR domain swaps on lupus-like disease.

## Results

### TLR779 recognizes RNA but signals through TLR9-TIR.

**Figure 1:**
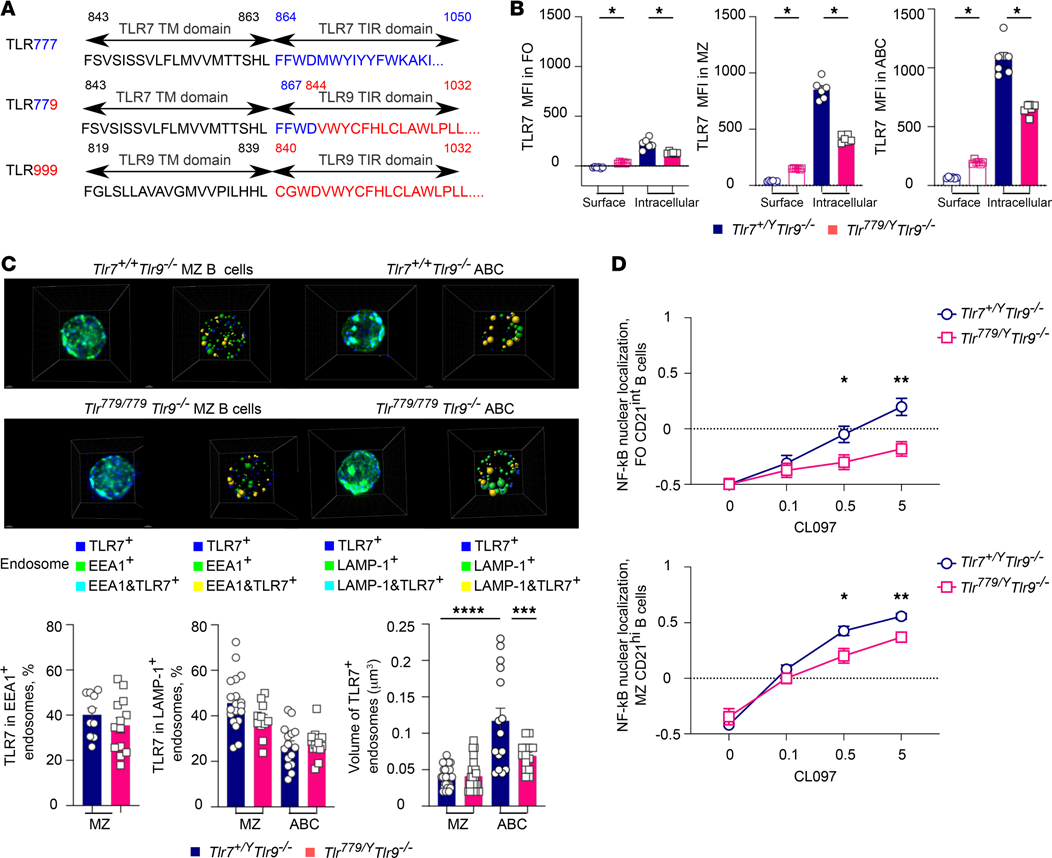
Creation of a chimeric RNA-sensing TLR7 with TLR9 signaling capacities. (**A**) Amino-acid sequences of the transmembrane-TIR junctions. (**B**) surface and intracellular staining of TLR7 in CD23^hi^ CD21^lo^ FO, CD23^lo^ CD21^hi^ MZ, CD11c^+^CD11b^+^ ABCs B cells of 5-week-old *Tlr7^+/Y^* or *Tlr^779/Y^* (all *Tlr*9^–/–^) male MRL/lpr mice. Data were analyzed by Wilcoxon matched-pairs signed rank test. (**C**) TLR7 endosomal localization was evaluated by confocal microscopy in sorted splenic MZ B cells and in ABCs of 6–7week old female *Tlr7^+/+^* and *Tlr^779/779^* MRL/lpr mice. Left and right panels show representative 1,000x magnification images and 3D reconstructed images of TLR7+ endosomes. Colored spheres indicate EEA1, LAMP-1, and TLR7 spot counting. The percentage of TLR7^+^ EEA1^+^ or LAMP-1^+^ endosomes in *Tlr7^+/+^*or *Tlr^779/779^* MZ B cells or ABCs (EEA1 staining, MZ B cells, *Tlr7^+/+^ n* = 10 cells, *Tlr^779/779^ n* = 13 cells; LAMP-1 staining, MZ B cells *Tlr7^+/+^n* = 20 cells, *Tlr^779/779^ n* = 11 cells; ABC *Tlr7^+/+^ n* = 15 cells, *Tlr^779/779^ n* = 14 cells; from 2 mice).The mean volume of the reconstructed TLR7 spots (MZ B cells, *Tlr7^+/+^ n* = 30 cells, *Tlr^779/779^ n =* 24 cells; ABC B cells, *Tlr7^+/+^ n =* 15 cells, *Tlr^779/779^ n =* 14 cells, from 2 mice) Data were analyzed by 1-way ANOVA with Sidak’s multiple comparisons test. (**D**) Splenocytes from 5–6-week-old *Tlr7^+/Y^ Tlr9^–/–^* or *Tlr^779/Y^ Tlr9^–/–^* male MRL/lpr mice were stimulated with different doses of TLR7 agonist (CL097; μg/ml) for 120 min. Quantification of the NF-κB nuclear localization score in FO CD21^int^ or MZ CD21^hi^ B cells (upper and lower panels). Data points indicate the mean score quantified for *n* = 6 mice per genotype and bars indicate the SEM of 2 experiments pooled. Data were analyzed by multiple paired *t* test. For statistics, *P < 0.05, **P < 0.01, ***P < 0.001, ****P < 0.0001.

We aligned the amino acid sequences of TLR7 and TLR9 transmembrane and TIR domains to generate a TLR^779^ construct, in which the TLR9 TIR domain is substituted into the TLR7 molecule (Figure 1A). The TLR molecules are described based on the source of their 3 functional domains: endosomal domain–transmembrane domain–signaling TIR domain. The resulting chimeric TLR779 binds RNA as WT TLR7 (TLR777), but signals like TLR9. To directly compare the functions of TLR777 and TLR779 in primary cells, we generated *Tlr^779/779^* MRL/lpr mice on a *Tlr9^–/–^* genetic background, such that the only TLR9 TIR domain present would be in the TLR779 molecule, thus rendering the source of TLR9-TIR signaling quality as unambiguous. We focused our characterization on B cells, as both TLR9 and TLR7 were demonstrated to exert their differential effects in B cells (7, 8). TLR779 was expressed in TLR7-expressing splenic B cells, i.e., marginal zone (MZ) B cells and CD11b^+^CD11c^+^ age-associated B cells (ABC), although at about half the amount of TLR777 (Figure 1B). There also was a very low level of surface expression of TLR779 but not TLR777 on B cells (Figure 1B).

As TLR endosomal localization could affect signaling (10, 12, 14), and as we have previously found that TLR7 and TLR9 are localized in different endosomes in naive B cells (9), we next checked the endosomal localization of TLR777 and TLR779 in MZ B cells and CD11b^+^CD11c^+^ ABCs of 6–7-week-old *Tlr9^–/–^* MRL/lpr mice (Figure 1C). In MZ B cells, TLR779 endosomal expression was consistent with the TLR7 endosomal pattern that we previously observed (9): TLR779 was located in small endosomes with a mean volume of 0.04 μm^3^, with 40% of them colocalizing with EEA1^+^ endosomes and 40% with LAMP-1^+^ endosomes (Figure 1C). These features were similar between *Tlr^779/779^* and *Tlr7^+/+^* MZ B cells (Figure 1C). In ABC, TLR777 was in larger endosomes with a mean volume of 0.12 μm^3^, a third of which were also LAMP-1^+^ (Figure 1C). Although TLR779 localization in ABC followed the same pattern, TLR779 endosomes were smaller compared with TLR777 endosomes (Figure 1C). This difference in endosome size, seen only in the ABCs, which are a postactivation cell type, is likely not a cell-intrinsic feature, and, rather, might reflect a change in the immune activation state of the animals. Owing to the much stronger TLR7-based signaling of *Tlr7^+/+^*, ABCs from this strain are likely to be more activated than ABCs from *Tlr^779/779^* mice, even at this early stage. As activation leads to endosomal and receptor remodeling and relocation, this could lead to the endosomal changes we observed. Collectively, these results show that substituting the TLR7-TIR with the TLR9-TIR domain did not detectably influence TLR779 endosomal localization.

To test if the substitution of the TLR9 TIR domain into TLR7 led to differences in signaling, we assessed NF-κB nuclear translocation (9) in *Tlr^779/Y^* or *Tlr7^+/Y^* (all *Tlr*9*^–/–^*) CD21^lo^ follicular (FO) and CD21^hi^ MZ splenic B cells after stimulation with graded doses of the TLR7 agonist (CL097). Consistent with our previous results that only approximately 18% of *Tlr7^+/Y^* FO B cells express TLR7 at low levels (9) (Figure 1B), NF-κB translocation upon stimulation with CL097 was lower in *Tlr7^+/Y^* FO compared with *Tlr7^+/Y^* MZ B cells (Figure 1D, blue curve upper and lower panels). Although NF-κB translocation in *Tlr^779/Y^* MZ B cell subsets was induced by CL097 stimulation, it was significantly reduced compared with *Tlr7^+/Y^* MZ B cell responses (Figure 1D). This result could reflect intrinsic differences of the TIR-signaling capacities, with TLR9 TIR mediating less overall signaling, as might be expected, and/or be due to the approximately 50% decrease in TLR779 expression compared with *Tlr7^+/Y^*.

**Figure 2:**
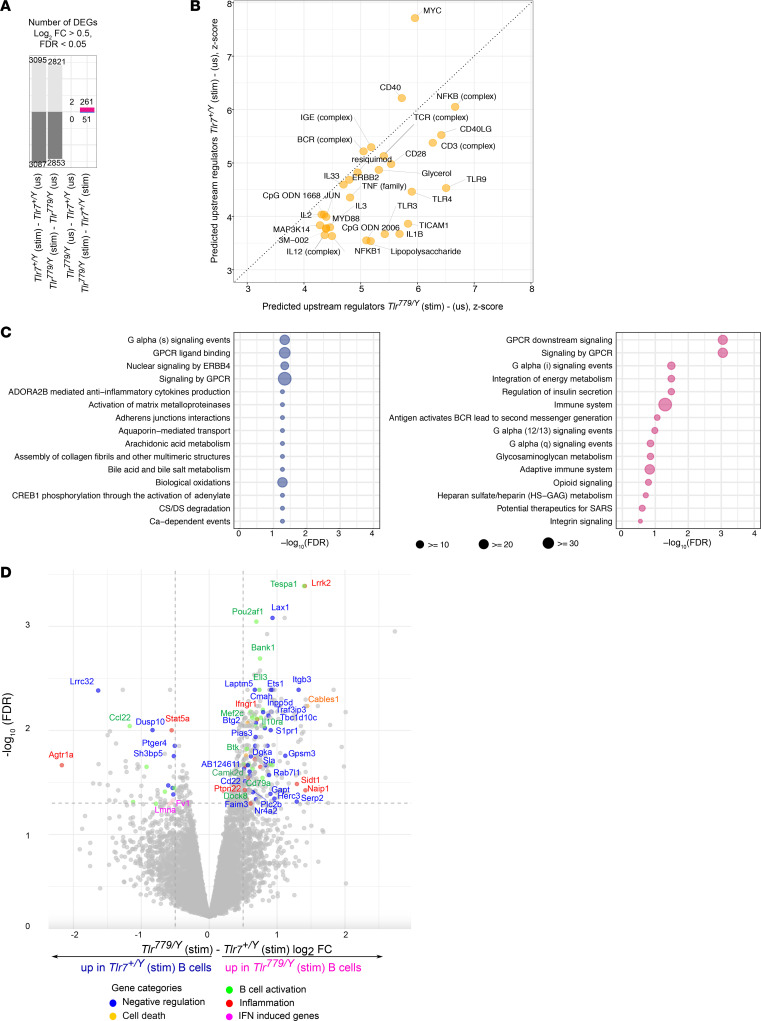
Differences in B cell–signaling qualities driven by TLR777 or TLR779. Transcriptome analysis of bead-purified B cells from 5–7week-old *Tlr7^+/Y^ Tlr9^–/–^* or *Tlr^779/Y^ Tlr9^–/–^* male MRL/lpr mice that were stimulated with TLR7 agonist (CL097; 5 μg/ml) for 4 hours. (**A**) Number of differentially expressed genes (DEGs) identified using the limma R package (log_2_ FC > 0.5 and FDR-corrected *P* < 0.05), between stimulated versus unstimulated samples (first 2 columns) or between the 2 genotypes, with or without stimulation (last 2 columns). (**B**) Upstream regulators that are predicted to be significantly activated upon CL097 stimulation by the IPA software in *Tlr7^+/Y^* (Y-axis) or *Tlr^779/Y^* (X-axis) B cells. XY plot shows the predicted z-score. (**C**) Bubble plot shows the top 15 reactome pathways enriched in *Tlr^779/Y^* (stim) (pink bubbles) and *Tlr7^+/Y^* (stim) (dark blue bubbles) regulated genes (from the *Tlr^779/Y^* (stim) vs *Tlr7^+/Y^* (stim) comparison). X-axis shows the –log_10_ FDR for the enriched terms displayed on Y-axis. Bubble size shows the genes in the pathway that are also differentially expressed in *Tlr^779/Y^* versus *Tlr7^+/Y^*. Enrichment was calculated using Fisher’s exact test (with all expressed genes as background) followed by Storey’s Q value FDR correction. (**D**) Volcano plot representing the DEGs between *Tlr7^+/Y^* and *Tlr^779/Y^* stimulated B cells. X-axis shows the log_2_ fold change value and Y-axis shows –log10 (FDR). The dotted lines separate the significant (FDR < 0.05) and nonsignificant (FDR > 0.05) genes and indicate the log_2_FC –0.5 and 0.5 cut-offs. The significant DEGs (log_2_ FC > 0.5, and FDR-corrected P value of < 0.05) were annotated based on the reported functions of their corresponding proteins in B cell activation (green dots), cell death (yellow dots), TLR-mediated inflammation (red dots), negative regulation of TLR-mediated inflammation and cell activation (blue dots), or if the genes were IFN-induced genes (pink dots).

To further investigate if the identity of the TIR domain influences the quality of signaling upon CL097 stimulation, we compared the transcriptomes of *Tlr^779/Y^* or *Tlr*7*^+/Y^* (all *Tlr*9*^–/–^*) purified B cells before and after stimulation with CL097 (Figure 2). There was no difference at basal state (before stimulation) between the 2 genotypes (Figure 2A, 3^rd^ column). Upon stimulation with CL097, *Tlr*^779/Y^ B cells demonstrated a similar number of significantly increased and decreased transcripts as for *Tlr*7*^+/Y^* B cells (around 3,000 genes in each direction; Figure 2A, first and second columns). The upstream regulators that were predicted to be activated upon stimulation with CL097 by the ingenuity pathway analysis (IPA) software were similar in both genotypes and included expected regulators such as MyD88, NF-κB, and TLR ligands (Figure 2B). These results indicate that TLR779 is clearly signaling competent. There were 312 differentially expressed genes (DEGs) between the B cells of the 2 genotypes after stimulation (Figure 2A, 4^th^ column). Interestingly, most of the DEGs were genes upregulated in *Tlr^779/Y^* B cells. These uniquely upregulated genes could reflect TLR9 TIR-specific properties (i.e., that would not be shared by TLR7-TIR). Most likely due to the relatively small number of DEGs observed, no clear biologically relevant pathway could be enriched from these differences using reactome pathway analysis (Figure 2C). Therefore, to assess the potential biological impact of the TIR domain substitution, we individually classified each significant DEG (log_2_FC > 0.5) based on annotated functions in B cells or TLR-mediated inflammation (Figure 2D). Interestingly, one third of the upregulated transcripts in *Tlr^779/Y^* B cells encoded for proteins that are reported to negatively regulate one of these functions, and, in particular, B cell activation (such as *Cd22, Faim3, Laptm5, Sla, Dgka, Tbc1d10c Inpp5d, Lax1, Traf3ip3*) and differentiation, such as *Ab124611, Btg2,* and *Ets1*. ETS1 is a transcription factor involved in the inhibition of antibody-secreting cell differentiation and autoimmune disease, which could participate in the protective and MyD88-independent function of TLR9 (9). The finding that TLR9 TIR mediates differential upregulation of these regulatory-associated genes is consistent with our previous published data suggesting that TLR9 has a protective signaling pathway induced by receptor ligation but independent of MyD88, which is presumably encoded by the TLR9-TIR domain (9).

### Tlr^779/779^ Tlr9^–/–^ MRL/lpr mice are protected from disease.

**Figure 3:**
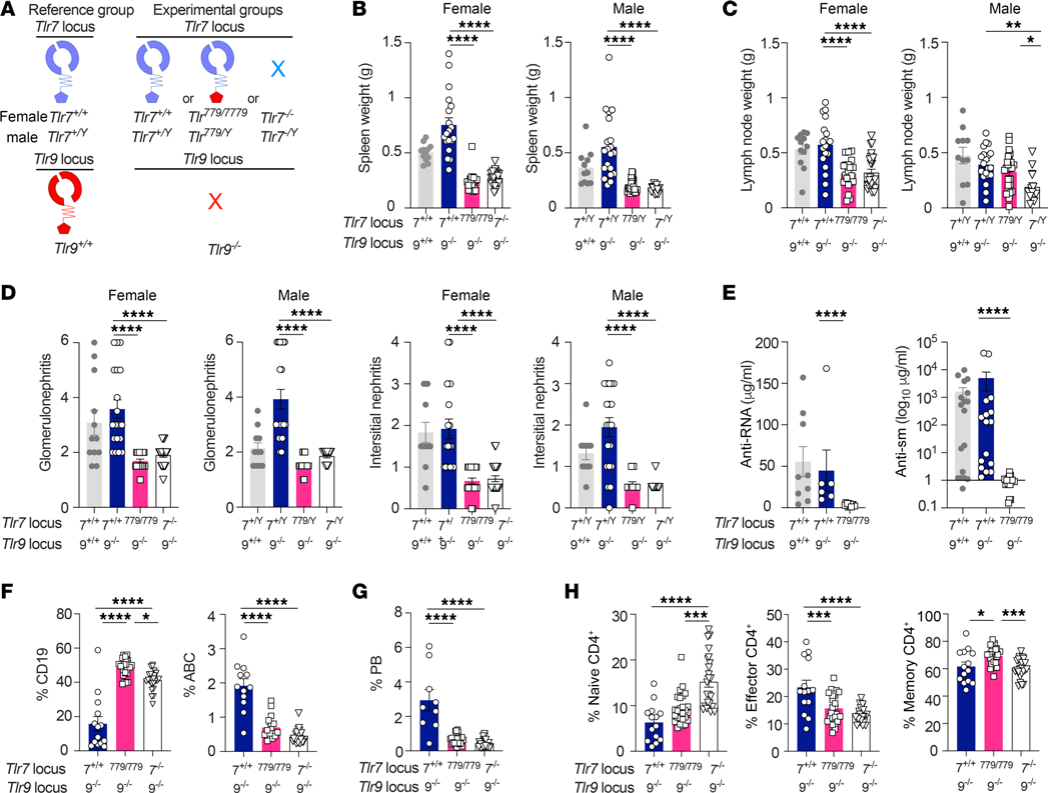
TLR^779^ protects from lupus disease. 16–18-week-old *Tlr7^+/+^*, *Tlr^779/779^* or *Tlr7^–/–^* (all *Tlr9*^–/–^) MRL/lpr mice were assessed for disease endpoints. Disease endpoints were also assessed in age-matched WT *Tlr7^+/+^* and *Tlr9^+/+^* MRL/lpr mice as a reference but were not included in the statistical analysis (**B**–**E**). (**A**) Schematic of the different mouse genotypes that are compared. The groups were labeled *Tlr7^+/+^, Tlr^779/779^* or *Tlr7^–/–^* if both males and females were included. (**B** and **C**) Spleen and lymph node weights were measured in mice of the indicated sex and genotypes. (**D**) Kidney pathology was assessed in mice of the indicated sex and genotypes. For **B–D**, reference group female *n =* 12, male *n* = 11; experimental group female *Tlr7^+/+^n* = 19, *Tlr^779/779^ n* = 21, *Tlr7^–/–^* *n =* 25; male *Tlr7^+/Y^n* = 20, *Tlr^779/Y^ n* = 24, *Tlr7^–/Y^ n* = 14. (**E**) Quantification of anti-RNA (reference group *n* = 9; experimental group *Tlr7^+/+^n* = 6, *Tlr^779/779^* *n* = 13, all females) and anti-Smith autoantibodies (reference group *n* = 18; experimental group *Tlr7^+/+^n* = 16, *Tlr^779/779^* *n* = 19, males and females). (**F**–**H**) Splenic B and T cell subsets were assessed by flow-cytometry in MRL/lpr mice of the indicated genotypes. (**F**) Percent of CD19^+^ cells among live splenocytes, and CD11b^+^ CD11c^+^ ABCs among live B cells (CD19^+^: *Tlr7^+/+^n* = 14, *Tlr^779/779^* *n* = 25, *Tlr7^–/–^* *n* = 24; ABC: *Tlr7^+/+^n* = 12, *Tlr^779/779^* *n* = 17, *Tlr*7^–/–^ *n* = 24). (**G**) Percent of TCR^–^CD44^hi^CD138^+^ plasmablasts among live splenocytes in mice of the indicated genotypes (*Tlr7^+/+^n* = 9, *Tlr^779/779^* *n* = 25, *Tlr7^–/–^* *n* = 24). (**H**) Percent of naive (CD62L^hi^ CD44^lo^), activated (CD62L^hi^ CD44^hi^), and memory (CD62L^lo^ CD44^hi^) T cells among live TCR^+^ CD4^+^ splenocytes (*Tlr7^+/+^n* = 14, *Tlr^779/779^* *n* = 25, *Tlr7^–/–^* *n* = 24). For all panels, data points indicate individual mice and bars indicate the mean ± SEM For statistics, *P < 0.05, **P < 0.01, ***P < 0.001, ****P < 0.0001, using a 1-way ANOVA with Tukey’s multiple comparisons test for all panels except **E**; Mann-Whitney test for panel **E**.

To determine if differences between signaling downstream of TLR779 and TLR777 affect disease severity in vivo, we assessed lupus-associated phenotypes in *Tlr7^+/+^ Tlr9^–/–^*, *Tlr^779/779^ Tlr9^–/–^* MRL/lpr mice (Figure 3A). We included *Tlr7^–/–^ Tlr9^–/–^* MRL/lpr mice as an additional control to compare the 50% decreased expression of TLR779 to a complete null allele, which would be the baseline of no influence for TLR7. Compared with *Tlr7^+/+^ Tlr9^–/–^* mice, *Tlr^779/779^ Tlr9^–/–^* mice demonstrated a dramatic amelioration of lupus disease, as evidenced by reduced splenomegaly, glomerulonephritis (GN) and interstitial nephritis (IN) scores (Figure 3, B–D). While both female and male *Tlr^779/779^* mice had markedly reduced lupus disease, lymphadenopathy reduction was only observed in female *Tlr^779/779^* mice, which also had a slight increase in TLR779 expression compared with male mice (Supplemental Figure 1A; supplemental material available online with this article; https://doi.org/10.1172/JCI189566DS1). Production of anti-RNA and anti-Smith autoantibodies was also abolished in *Tlr*^779/779^*Tlr*9^–/–^ mice (Figure 3E). In fact, *Tlr^779/779^Tlr*9*^–/–^* mice displayed the same level of reduced disease as the fully TLR-deficient *Tlr7^–/–^Tlr*9*^–/–^* MRL/lpr mice (Figure 3, B–D). When compared with *Tlr*7*^+/+^Tlr*9*^–/–^* mice (Figure 3F and Supplemental Figure 1B), both *Tlr^779/779^Tlr*9*^–/–^* and *Tlr7^–/–^Tlr*9*^–/–^* mice had an increase in percentages of total splenic CD19^+^ and MZ B cells; decreased proportions of pathogenic ABCs, including the CD138^+^subsets (9) (Supplemental Figure 1C); and decreased frequencies of plasmablasts (Figure 3G). Nonetheless, the phenotype of the *Tlr^779/779^Tlr*9*^–/–^* mice was distinct from the *Tlr7^–/–^Tlr*9*^–/–^* mice in terms of immune activation: *Tlr^779/779^Tlr*9*^–/–^* mice had intermediate levels of the CD11c^+^CD19^+^ subset of ABC B cells (15), GC-like B cells (Supplemental Figure 1B), and of naive and memory subsets of CD4^+^ and CD8^+^ T cells (Figure 3H and Supplemental Figure 1D), compared with the diseased *Tlr7^+/+^ Tlr9^–/–^* mice and the fully protected *Tlr7^–/–^ Tlr9^–/–^* mice. This result confirms that TLR779 is not simply a “null” mutant in vivo, in line with its preserved signaling activity in vitro as well as the robust modulation of gene expression that this protein mediates (Figures 1 and 2). Hence, we conclude that either TLR7 TIR has a substantially more proinflammatory signaling quality than TLR9-TIR, and/ or that TLR9-TIR has a dominant protective effect that counterbalances its own proinflammatory MyD88-driven signaling. The latter interpretation is favored as it also is supported by TLR9 point mutants that fail to conduct MyD88-dependent signaling yet that protect from disease (9). Nonetheless, these data do not rule out a role of the decreased in TLR779 expression in contributing to protection.

### The chimeric TLR997 recognizes DNA but signals through TLR7-TIR.

**Figure 4:**
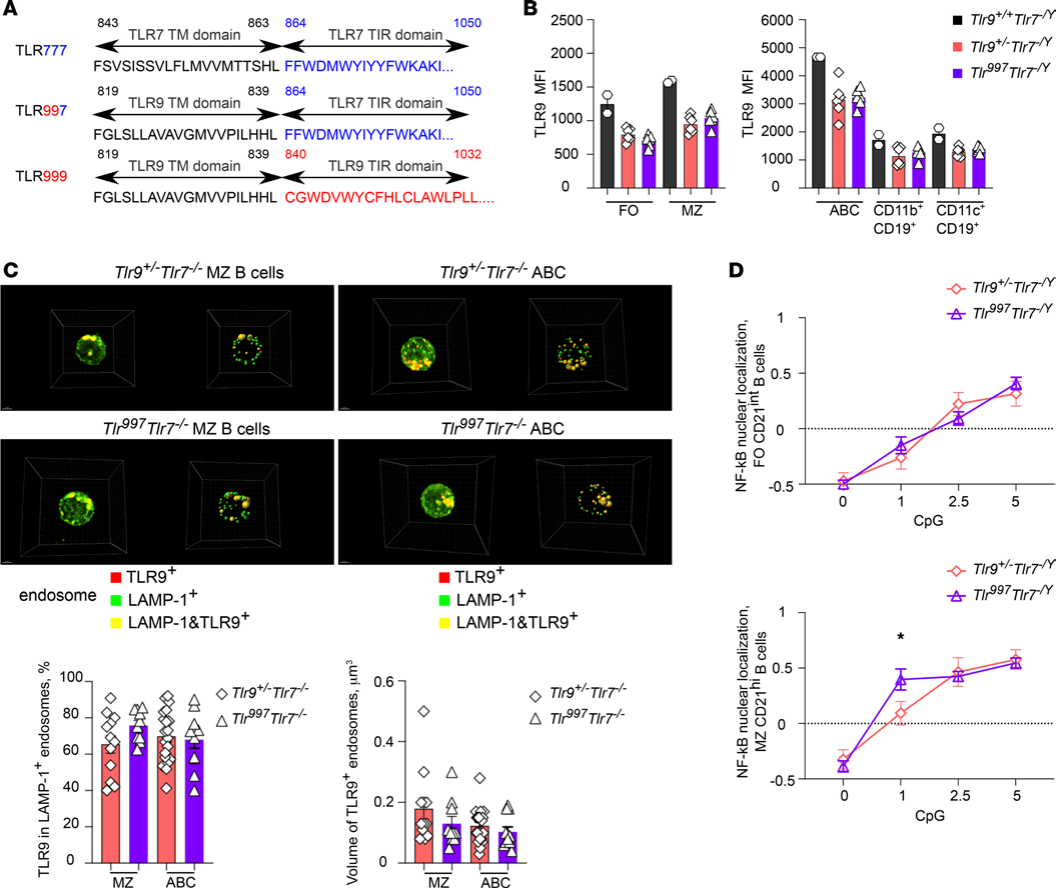
Creation of a chimeric DNA-sensing TLR9 that signals through TLR7-TIR signaling domain. (**A**) Amino-acid sequences of the transmembrane-TIR junctions of TLR999, TLR777, and the TLR997 mutant. The TLR molecules are described based on the source of their 3 functional domains: endosomal domain–transmembrane domain–signaling TIR domain. (**B**) intracellular staining of TLR9 in FO, MZ, CD11c^+^CD11b^+^ ABCs, CD11b^+^, and CD11c^+^ B cells of 5–7-week-old *Tlr9^+/+^*, *Tlr9^+/–^*, or *Tlr^997^* (all *Tlr7^–/Y^*) male MRL/lpr mice. Data points indicate individual mice (*n* = 6 per genotype and bars indicate the mean ± SEM of 2 pooled experiments, except for *Tlr9^+/+^*, *n* = 2 mice). (**C**) TLR9 endosomal localization in flow-sorted splenic CD21^hi^ MZ (left column) and CD11c^+^CD11b^+^ ABC (right column) B cells of 6–7 week old *Tlr9^+/–^* or *Tlr^997^* (all *Tlr7^–/–^*) female MRL/lpr mice was evaluated by confocal microscopy. Images were acquired at 1,000x magnification. Representative images of TLR9^+^ endosomes were made with 3D reconstruction (left panels). Colored spheres indicate LAMP1 and TLR9 spot counting generated from confocal images (right panels). The percentage of TLR9^+^ LAMP-1^+^ endosomes and the mean volume of the reconstructed TLR9 spots were measured using the Imaris software in *Tlr9^+/–^* or *Tlr^997^* MZ and ABC B cells. (MZ B cells, *Tlr9^+/–^ n =* 12 cells, *Tlr^997^ n* = 10 cells; ABC B cells, *Tlr9^+/–^ n* = 20 cells, *Tlr^997^ n* = 11 cells, from 2 mice per genotype). (**D**) Splenocytes from 5–7-week-old *Tlr9^+/–^ Tlr7^–/Y^* or *Tlr^997^ Tlr7^–/Y^* male MRL/lpr mice were stimulated with different doses of TLR9 agonist (CpG, μg/ml) for 120 min. Quantification of the NF-κB nuclear localization score in FO CD21^int^ or MZ CD21^hi^ B cells (upper and lower panels). Data points indicate the mean score quantified for *n* = 6 mice per genotype and bars indicate the SEM of 2 experiments pooled. *P < 0.05, **P < 0.01, ***P < 0.001, ****P < 0.0001. Data between the 2 genotypes were analyzed by multiple paired *t* test.

If the ameliorated disease in *Tlr^779/779^ Tlr9^–/–^* mice was due to the TLR9 TIR domain possessing more regulatory and less proinflammatory function compared with the TLR7 TIR domain, then reciprocal swapping of the TLR7 TIR domain onto TLR9 should promote disease, representing a critical test of the model. To probe this, we created TLR997 by replacing TLR9-TIR by TLR7-TIR in TLR9 (Figure 4A). To compare the functions of TLR9-TIR and TLR7-TIR in primary cells, we inserted *Tlr^997^* into the endogenous *Tlr9* locus of MRL/lpr mice, using CRISPR/Cas9, and crossed the resultant allele to *Tlr7*^–/–^ MRL/lpr mice to generate *Tlr^997/997^* homozygous *Tlr7^–/–^* MRL/lpr mice (referred to below as *Tlr^997^ Tlr7^–/–^* mice). In *Tlr^997^ Tlr7^–/–^* B cells, the only source of TLR7-TIR “quality of signaling” comes from TLR997. Empirically, we found that *Tlr^997^ Tlr7*^–/–^ B cell subsets had 50% of WT amounts of TLR9 protein (Figure 4B). Thus, we controlled for overall TLR9 expression by comparing *Tlr^997^ Tlr7^–/–^* B cells to *Tlr*9*^+/–^ Tlr7^–/–^* B cells, which express an equal amount of TLR9 (Figure 4B). Importantly, endosomal localization of TLR997 in MZ B cells and ABCs was consistent with a “TLR9 pattern” (i.e. located in larger endosomes, with a mean volume of 0.13–0.18μm^3^, which were also LAMP-1^+^ (9)) and was comparable to TLR999 endosomal localization (Figure 4C). Collectively, these results show that, in terms of expression and localization, there are no detectable differences between *Tlr^997^* and *Tlr9^+/–^* B cells, while they do differ in the nature of the TIR domain that their TLR9 molecule harbors.

To test if there were differences in NF-κB signaling thresholds driven by TLR997 or TLR999 in B cells, we titrated CpG DNA doses in *Tlr9*^+/+^ BALB/c B cells to identify a dose range that would cover the full spectrum of B cell activation (Supplemental Figure 2A). CD21^hi^ MZ B cells had a lower CpG signaling threshold than CD21^int^ FO B cells. Because 1μg/mL of CpG gave no detectable response, while 2.5 gave intermediate and 5 μg/mL gave nearly maximal responses, we chose these 3 doses to cover the threshold response dose range for comparing the *Tlr9^+/–^* and *Tlr^997/997^* B cell activation profile. The response was indistinguishable in FO B cells, which express relatively lower amounts of TLR9. *Tlr^997^* *Tlr7^–/Y^* MZ B cells, which express more TLR9, showed an enhanced response to a lower dose of CpG DNA (1 μg/mL) compared with *Tlr9^+/–^ Tlr7^–/Y^* CD21^hi^ (MZ) B cells (Figure 4D). Indeed, stimulation with CpG at 1 μg/mL yielded marginal NF-κB nuclear translocation in *Tlr^999^* MZ B cells (as expected based on the BALB/c data) whereas *Tlr^997^* MZ B cells were fully activated. In accordance with the BALB/c data, *Tlr9^+/–^* *Tlr7^–/Y^* CD21^hi^ MZ B cells had a lower threshold for response to CpG compared with *Tlr9^+/–^ Tlr7^–/Y^* CD21^int^ FO B cells, confirming that there is a subset specificity to the CpG response (Figure 4D, red curve upper and lower panels), and potentially explaining the reproducible increased sensitivity of B cells of the *Tlr^997^* *Tlr7^–/Y^* genotype only in MZ B cells. The increased sensitivity to lower doses of CpG is consistent with the notion that TLR7 TIR may transduce a stronger signal than TLR9 TIR, as also indicated by the data from the *Tlr^779/779^* B cells shown above (Figure 1).

**Figure 5:**
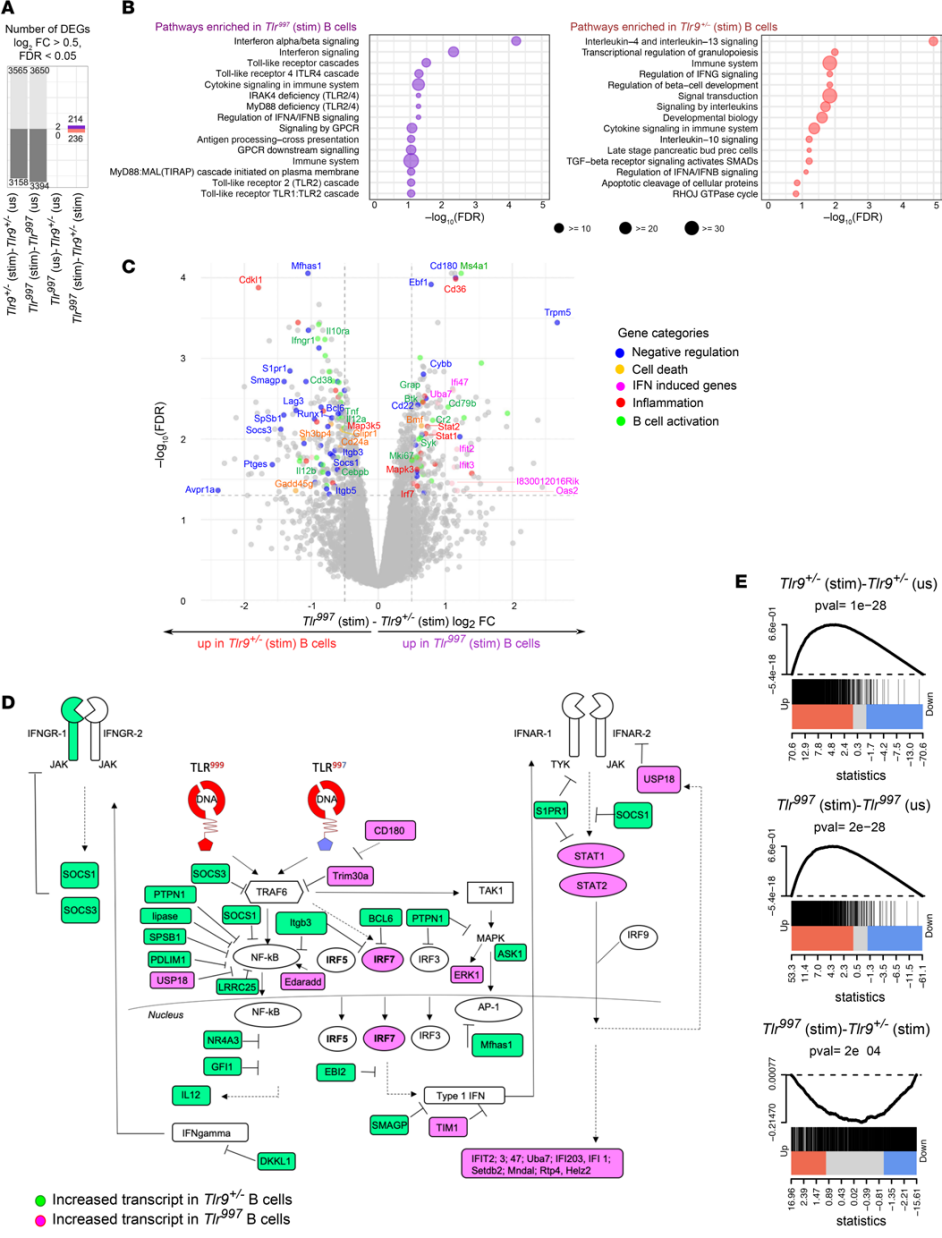
Differences in B cell signaling qualities driven by TLR999 or TLR997. Transcriptome analysis of bead-purified B cells from 5-week-old *Tlr9^+/–^ Tlr7^–/–^* or *Tlr^997^ Tlr7^–/–^* female MRL/lpr mice that were stimulated with TLR9 agonist (CpG, 10 μg/ml) for 4 hours. (**A**) Number of differentially expressed genes (DEGs) identified using the limma R package (log_2_ FC > 0.5, and FDR-corrected *P* value of < 0.05). (**B**) Bubble plots show the top 15 reactome pathways enriched in *Tlr^997^* (purple bubbles) and *Tlr9^+/–^* (salmon bubbles) regulated genes from the *Tlr^997^* (stim) vs *Tlr9*^+/–^ (stim) comparison. Bubble size reflects the number of genes in the pathway that are also differentially expressed in *Tlr^997^* vs *Tlr9^+/–^*. Enrichment was calculated using Fisher’s exact test (with all expressed genes as background) followed by Storey’s Q value FDR correction. (**C**) Volcano plot representing the DEGs between *Tlr^997^* and *Tlr9^+/–^* CpG-stimulated B cells. The significant DEGs (log_2_ FC > 0.5) were annotated based on the reported functions of their corresponding proteins in B cell activation (green dots), cell death (yellow dots), TLR-mediated inflammation (red dots), negative regulation of TLR-mediated inflammation and/or B cell activation (blue dots), or if the genes were IFN-induced genes (pink dots). (**D**) Diagram depicting how proteins encoded by the curated DEGs in **C** could promote or regulate NF-κB, IRF, MAPK, IFN type 1 or 2 signaling pathways. In the cartoons of the chimeric TLR, TLR9-driven domains are in red and TLR7-derived domain in blue. (**E**) A TLR9-induced gene set signature for B cells of *Tlr9^+/+^* BALB/c mice was generated. It comprises 1,724 upregulated genes (log_2_FC > 1 and FDR P value < 0.05) after a 4-hour in vitro CpG stimulation compared with unstimulated cells. Enrichment of this TLR9-induced gene set was assessed before and after TLR9 stimulation in *Tlr9^+/–^* B cells (left panel) and in *Tlr^997^* (middle panel) B cells and between *Tlr9^+/–^ and Tlr^997^* stimulated B cells (right panel). The *P* value was calculated using the rankSumTestWithCorrelation function from the R limma package.

To test if CpG stimulation of B cells via TLR999 or TLR997 resulted in qualitatively different responses, we compared the transcriptomes of resting and of CpG-stimulated *Tlr^997^* and *Tlr9^+/–^* (all *Tlr7^–/–^*) B cells by RNA-seq. Whereas there was no transcriptome difference at baseline (Figure 5A, third column), after stimulation both genotypes upregulated and downregulated a large and similar number of transcripts (over 3,000 in each direction for each genotype), reflective of the signaling competency of the chimeric TLR9 (Figure 5A). There were 450 DEGs between *Tlr^997^* and *Tlr9^+/–^* (all *Tlr7^–/–^*) B cells after stimulation (Figure 5A, fourth column). Interestingly, the top 2 pathways enriched in *Tlr^997^* *Tlr7^–/–^* B cells were “type 1 IFN signaling” and “IFN signaling pathways”, suggesting that TLR7-TIR is more proinflammatory (Figure 5B). In *Tlr9^+/–^* *Tlr7^–/–^* B cells, IL-4 and IL-13 signaling, IL-10 and TGF-β signaling pathways were significantly enriched (Figure 5B), that may be indicative of regulatory or “type 2” responses.

Even though statistically significant, only a small number of DEGs in each pathway was identified above by unsupervised analysis. Thus, we annotated each significant DEG (log_2_FC > 0.5) for known functions in B cell activation, TLR-mediated inflammation, negative regulation of either of these functions, or cell death (Figure 5C). To put these data in context, we constructed a diagram representing how the proteins encoded by these DEG would promote or regulate signaling pathways downstream of TLR stimulation (Figure 5D). Consistent with our previous published data and the *Tlr^779/779^* B cell transcriptome (Figure 2), in *Tlr9^+/–^* compared with *Tlr^997^* stimulated B cells we found more transcripts encoding for proteins that could inhibit NF-κB, IRF, MAPK, and IFN type 1 or 2 signaling pathways (Figure 5, C and D). These proteins could regulate downstream signaling by several mechanisms. BCL6 binds to the *Irf7* locus and represses its transcription (16), while GFI1 interacts with p65 in the nucleus and blocks p65 target gene promoters (17). SOCS1 and SOCS3, transcripts for which are upregulated in *Tlr9^+/–^* B cells, negatively regulate cytokine-stimulated STAT pathways as well as IRF7 (18, 19). RUNX1 interacts with IKKa/IKKb, blocking their phosphorylation (20). PTPN1B negatively regulates multiple Ser-Thr kinase pathways, for example dephosphorylating P-p38 (21, 22). PDLIM1 sequesters p65 in the cytoplasm (23). S1PR1 promotes IFNAR1 degradation in pDC (24) and similarly LRRC25 promotes p65 degradation (25). Integrins β 3 and 5 control trafficking to lysosomes, where signaling is terminated (26). Interestingly, some of the aforementioned transcripts, such as *Itgb3* and *S1pr1*, were upregulated in both *Tlr9*^+/–^ B cells compared with *Tlr^997^* B cells and *Tlr^779/Y^* B cells in comparison with *Tlr7^+/Y^* B cells after respective ligand stimulation, suggesting that TIR domains, regardless of the ligand, may control their expression.

We also sought a “TLR9-TIR” gene expression signature, which in principle could be extracted by overlapping gene expression downstream of signaling via the 2 TLR9-TIR–containing molecules, TLR779 and TLR999. However, this approach would be hard to interpret because of the many differences between TLR779 and TLR999: TLR779 is X linked, has IFN-induced transcription, is localized to TLR7 endosomes, and recognizes RNA ligands in certain B cell subsets (MZ and ABC) whereas TLR999 is autosomal and constitutively transcribed, recognizes DNA, and is localized in LAMP-1 endosomes in all B cell subsets. Instead, we reasoned that if there were a “TLR9-TIR signature”, it should be lost to some extent in *Tlr*^997^ CpG-stimulated B cells compared with *Tlr*^999^ CpG-stimulated B cells. Therefore, to establish a baseline for TLR9-specific gene induction, we generated TLR9-induced gene set signatures for *Tlr9^+/+^* B cells in BALB/c mice. These were comprised of genes with significantly increased expression (log_2_FC > 1, FDR < 0.05) after CpG stimulation, respectively, compared with the corresponding unstimulated cells. As expected, the TLR9 gene signature was highly enriched in both *Tlr9^+/–^* and *Tlr^997^* CpG-stimulated B cells, compared with the corresponding nonstimulated B cells (Figure 5E). Critically, though, the TLR9 gene signature was significantly enriched in CpG-stimulated *Tlr9^+/–^* compared with *Tlr^997^* B cells (Figure 5E). Hence, the TLR9 TIR domain is required to mediate the TLR9-like “quality” of downstream gene induction, while the TLR7 TIR domain does not equally do this. Taken together, these patterns suggest that the TIR domain intrinsically controls the quality of the genes induced, whether for TLR7 or TLR9.

### TLR997 and TLR999 differentially impact B cell activation.

**Figure 6:**
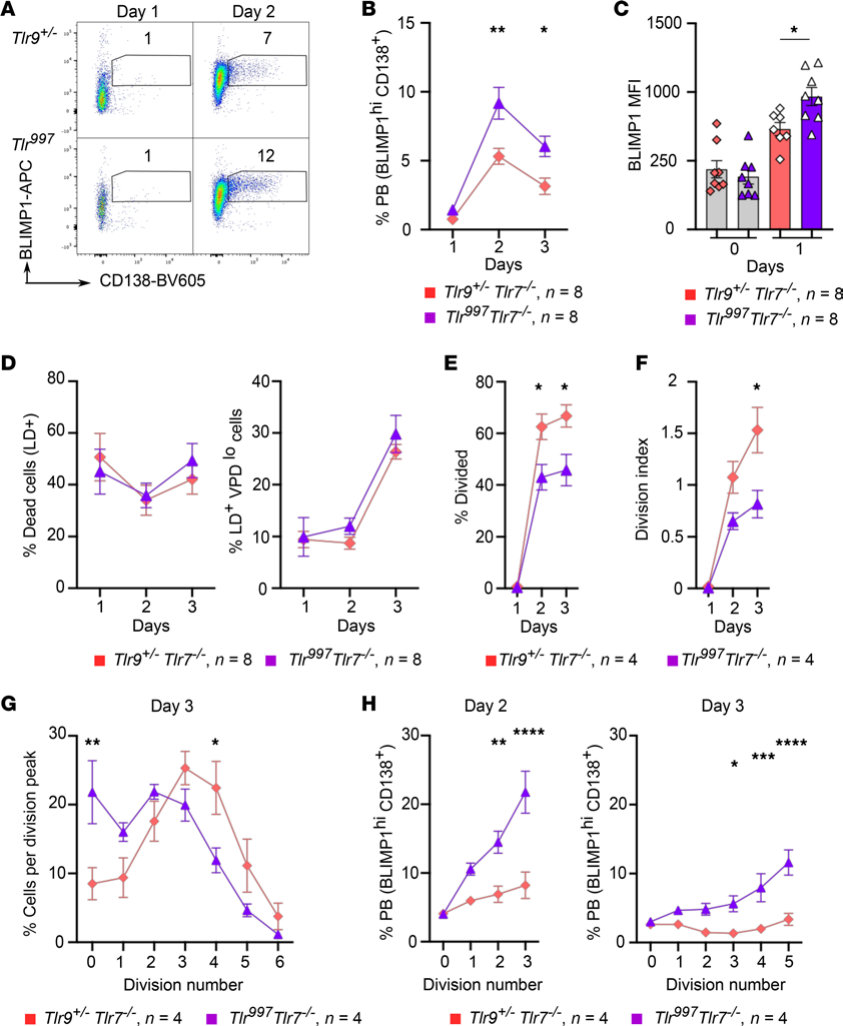
TLR997 and TLR999 differentially impact B cell differentiation and proliferation. B cells from 5–7-week-old *Tlr9^+/–^* or *Tlr^997^* (all *Tlr7^–/–^*) MRL/lpr mice were labeled with violet proliferation dye (VPD) and cultured for 1, 2, or 3 days with CpG (1 μg/ml). (**A**) Representative flow cytometry plots gated on live B cells. (**B**) Quantification of BLIMP1^hi^CD138^+^ plasmablasts (PB) among live B cells. One-way ANOVA with Sidak’s multiple comparisons test was used to compare both genotypes. (**C**) BLIMP1 MFI in live B cells. Symbols indicate individual mice and error bars represent SEM. An unpaired *t* test was used to compare both genotypes at day 1. (**D**) Percentage of live-dead dye positive (LD^+^) and LD^+^VPD^lo^ cells (which correspond to post-proliferative dead cells). For panels **E**–**H**, due to batch effects that led to differences in the overall B cell proliferation profiles, results from experiments 1 and 2 (shown in **E**–**H**) and experiments 3 and 4 (shown in Supplemental Figure 3) were analyzed separately. (**E**) Percentage of live B cells that divided. (**F**) The FlowJo Proliferation Platform analysis was used to determine the division index. (**G**) Cell divisions were gated based on each proliferation peak of live B cells. Division 0 corresponds to undivided cells. Y-axis shows the percentage of total live B cells within each division number at day 3 (61). (**H**) The percentage of PB for each division number was plotted. For panels **E**–**H**, symbols indicate mean and error bars are the SEM from *n* = 4 mice per genotype derived from 2 experiments. For **E** and **F**, 1-way ANOVA with Sidak’s multiple comparisons test was used. *P < 0.05, **P < 0.01, ***P < 0.001, ****P < 0.0001.

To assess whether B cell–intrinsic qualitative differences driven by TLR997 and TLR999 impact B cell functions in vitro*,* we compared *Tlr9^+/–^* with *Tlr^997^* (all *Tlr7^–/–^*) B cell activation after CpG DNA stimulation. CpG DNA stimulation induced BLIMP1^hi^ CD138^+^ plasmablast differentiation in both genotypes of B cells, which was maximal at day 2 of culture (Figure 6, A and B). However, *Tlr^997^* B cells yielded a significantly higher fraction of plasmablasts at day 2, which was also seen at day 3 (Figure 6, A and B). This nearly 2-fold increase could have been attributed to B-cell–intrinsic differences in mortality, proliferation, and/or differentiation, which we further assessed by flow cytometry. *Tlr9^+/–^* B cells demonstrated lower levels of BLIMP1 induction compared with *Tlr^997^* B cells at day 1, just before many fully differentiated plasmablasts could be observed, suggesting that TLR7-TIR promotes more differentiation compared with the TLR9-TIR (Figure 6C). In contrast, there was no difference in cell death between *Tlr^997^* and *Tlr9^+/–^* B cells at all tested time points after CpG DNA stimulation (Figure 6D and Supplemental Figure 3A). Although CpG DNA promoted B cell proliferation, there were batch effects between our experimental replicates with differences in the proportion of proliferating B cells and division numbers, requiring us to analyze the data separately (Supplemental Figure 3B). Nonetheless, in all cases, *Tlr9^+/–^* B cells proliferated more than *Tlr^997^* B cells at days 2 and 3, when evaluated by the proportion of total live B cells that have divided at least once, cells per division peak, or the division index (Figure 6 E–G, and Supplemental Figure 3, C–E). While the BLIMP1^hi^ CD138^+^ *Tlr^997^* plasmablast proportion increased proportionally with the number of cell divisions, proving that *Tlr^997^* B cells proliferate and differentiate, the proportion of *Tlr9^+/–^* plasmablasts did not increase with division to nearly the same extent as observed in the *Tlr^997^* cultures, further documenting that *Tlr9^+/–^* differentiate less even while dividing to an even greater extent (Figure 6H and Supplemental Figure 3F). Altogether, these data suggest that TLR9 and TLR7 TIRs mediate qualitatively and quantitatively different responses to ligand activation; TLR9-TIR predominantly promotes B cell proliferation, while TLR7-TIR promotes plasmablast differentiation.

### TLR997 exacerbates lupus disease.

**Figure 7:**
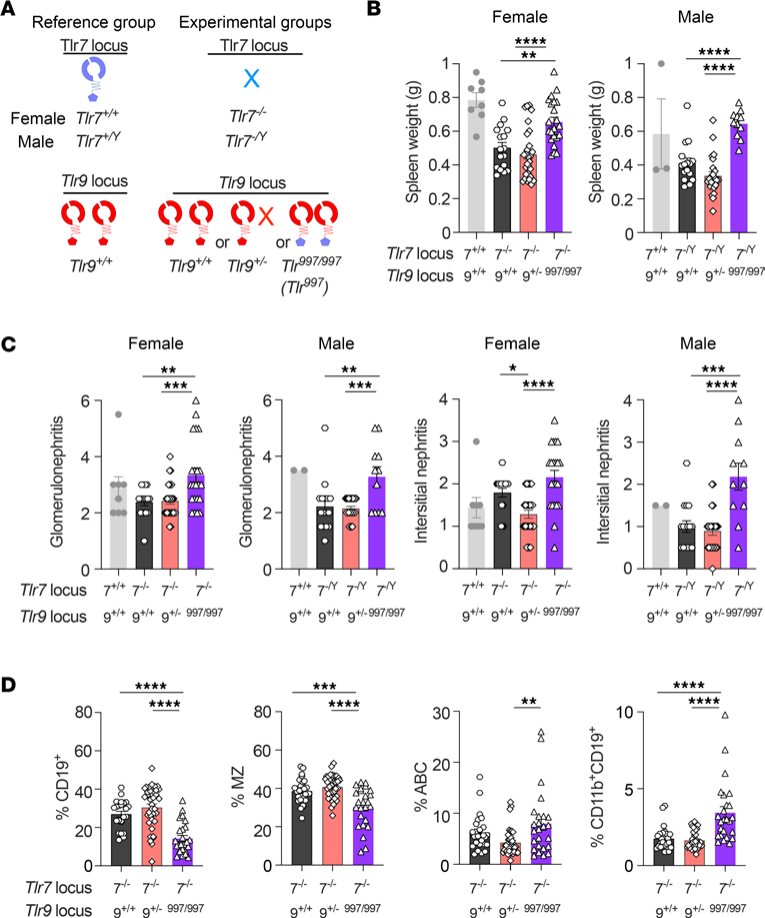
TLR997 exacerbates lupus disease. 18–21-week-old *Tlr9^+/+^*, *Tlr9^+/–^* or *Tlr^997/997^* (referred to as *Tlr^997^*) (all *Tlr7^–/–^*) MRL/lpr mice were assessed for disease endpoints. Disease endpoints were also assessed in 18–20-week-old WT *Tlr7^+/+^* and *Tlr9^+/+^* MRL/lpr mice as a reference but were not included in the statistical analysis (**B** and **C**). (**A**) Schematic of the different mouse genotypes that are compared. (**B**) Spleen weights were measured in mice of the indicated gender and genotypes. (**C**) Kidney pathology was assessed in mice of the indicated gender and genotypes. (For **B** and **C**, female *Tlr9^+/+^n* = 17, *Tlr9^+/–^ n* = 28, *Tlr^997^* *n* = 22, *Tlr9^+/+^ Tlr7^+/+^ n* = 8; male *Tlr9^+/+^n* = 16, *Tlr9^+/–^ n* = 24, *Tlr^997^ n* = 11, *Tlr9^+/+^ Tlr7^+/Y^ n* = 2 or 3) (**D**) Splenic B cell subsets were assessed by flow cytometry in mice of the indicated genotypes. Percent of CD19^+^ cells among live splenocytes, and percent of CD23^lo^ CD21^hi^ MZ, CD11b^+^ CD11c^+^ ABCs and CD11b^+^ cells among live B cells (*Tlr9^+/+^* *n* = 26, *Tlr9^+/–^ n* = 36, *Tlr^997^* *n* = 25). For all panels, data points indicate individual mice and bars indicate the mean ± SEM. For statistics, *P < 0.05, **P < 0.01, ****P < 0.001, ****P < 0.0001, 1-way ANOVA with Tukey’s multiple comparisons test.

To test if replacement of the TLR9 TIR domain with the TLR7 TIR domain in the *Tlr9* locus impacts disease, we evaluated *Tlr9^+/+^*, *Tlr9^+/–^*, and *Tlr^997^* MRL/lpr mice (all *Tlr7^–/–^*) at disease endpoints (Figure 7A). There were no significant differences in disease severity between *Tlr9^+/+^* and *Tlr9^+/–^* mice (Figure 7, B and C). However, compared with *Tlr9^+/–^Tlr7^–/–^* MRL/lpr mice, *Tlr^997^Tlr7^–/–^* mice had markedly exacerbated lupus disease, with increased spleen weight (Figure 7B) and higher glomerulonephritis and interstitial nephritis scores (Figure 7C). Disease exacerbation in *Tlr^997^Tlr7^–/–^* mice was similar in both males and females (Figure 7, B and C, and Supplemental Figure 4A). Interestingly, though, there was no difference in amounts of serum anti-nucleosome autoantibodies, consistent with prior evidence that disease and anti-nucleosome antibodies are unlinked (9, 27, 28) (Supplemental Figure 4B). As expected, given that all mice lacked TLR ectodomains that can recognize RNA, the levels of anti-Smith and anti-RNA autoantibodies were very low (Supplemental Figure 4B). Commensurate with substantially greater target tissue damage, *Tlr^997^Tlr7^–/–^* mice had increased percentages of autoreactive B cell subsets, such as ABC and CD11b^+^CD19^+^ B cells, and lower frequencies of normal MZ and FO B cells compared with *Tlr9^+/–^* *Tlr7^–/–^* mice (Figure 7D and Supplemental Figure 4C). There were few differences in T cell activation, except that *Tlr^997^Tlr7^–/–^* mice had fewer naive T cells than *Tlr9^+/–^* *Tlr7^–/–^* control mice, again consistent with increased disease mediated by the expression of the TLR7 TIR domain (Supplemental Figure 4D).

## Discussion

Here, we used a genetic approach to gain insight into the basis for the opposing functional properties of TLR7 and TLR9, a paradox that first came to light almost 20 years ago and has remained unsolved, due, in large part, to the complexity of the problem. In principle, the different functional roles of TLR7 and TLR9 could be attributed to differences in the nature of their ligands, receptor expression patterns, and/or downstream signaling outputs. While known differences in the availability and in vivo turnover of RNA versus DNA (29) along with the fact that TLR7 is more inducible by inflammatory signals (30, 31) are not excluded as contributing, these factors are controlled in our study. Thus, our data strongly implicate fundamental differences in TLR signaling per se, encoded by TIR domains, as a major and previously undocumented cause; most reference sources have implicitly assumed equivalent TIR domain function.

In this report, we, however, show that reciprocal genetic swaps of TLR7 and TLR9 TIR domains map inflammatory versus regulatory activity to TLR7 versus TLR9 TIR domains, respectively, regardless of ligand specificity and locus-specific expression patterns. The TLR9 TIR domain manifests regulatory activity — whether linked to the TLR9 ectodomain, as previously established (6, 9), or the TLR7 ectodomain and locus-specific expression pattern, as demonstrated here by the *Tlr^779^* mutant. Conversely, the TLR7 TIR domain manifests proinflammatory activity, whether linked to the TLR7 ectodomain (3, 4, 6), as previously shown by both us and others, or the TLR9 ectodomain and locus-specific expression pattern, as demonstrated here by the *Tlr^997^* mutant. Moreover, the substantial differences between the TIR domains in terms of downstream gene transcript induction upon ligand stimulation in vitro further support the conclusion that TLR7 and TLR9 TIRs are not equivalent. In fact, the genes that are differentially induced, particularly in the comparison between TLR999 and TLR997, support a more proinflammatory role for the TLR7 TIR domain compared with the TLR9 TIR domain, with IFN-stimulated pathways being enriched in the context of the TLR7 TIR domain.

An imbalance of TLR7 and TLR9 trafficking, induced by a mutation of their shared chaperone Unc93b, leads to increased TLR7 expression and signaling, which caused a lupus-like disease in mice (32, 33). Recently, several mutations of the human Unc93b that enhance TLR7 but not TLR9 activity were shown to mediate a rare form of genetically mediated pediatric lupus (34–37). These important studies provided evidence that enhanced TLR7 signaling could drive lupus, and mechanisms by which more TLR7 protein could be expressed. However, they did not elucidate why TLR7 and not TLR9 can mediate this effect. Our results add new insights into why “more TLR7/less TLR9” could be pathogenic in these situations: TLR7 and TLR9 signaling qualities are not equivalent, with TLR7 being more pro- and TLR9 more antiinflammatory. It is also important to note that other differences between intact TLR7 and TLR9, apart from TIR domain-encoded aspects, undoubtedly also play important roles. These could include differences in intracellular trafficking, encoded by transmembrane domains, which could lead to differential signaling (29, 38–42); differences in ligand abundance and location and hence ligand-dependent activation; as well as transcriptional induction differences mediated by the cis-acting elements in the distinct genomic loci of *Tlr7* and *Tlr9*.

The disparate disease outcomes and downstream gene expression patterns raise the question of how the 2 TIR domains and TLRs function to achieve these divergent outcomes. Trafficking plays a role in TLR7 versus TLR9 signaling (14), but is unlikely to explain the current findings, as TLR7/9 chimeric molecules trafficked like the parent molecules, yet signaled very differently. Similar localization of the chimeric molecules is expected, since TM domains are thought to control trafficking of endosomal TLRs, and these remained parental type. Indeed, CryoEM studies have shown that Unc93b, the key chaperone that stabilizes TLR expression (43) and initiates trafficking of both TLR7 and TLR9, binds to TLRs via the transmembrane and luminal juxtamembrane surfaces (13). Thus, even though differences in binding and regulation between Unc93b and the 2 TLRs has been reported (33, 39, 44), swapping TIR domains is unlikely to change their Unc93b interactions. Further, differential Unc93b binding would mediate different amounts of TLR7/9 maturation, but would not affect signal quality per se; whereas, we were able to match amounts of WT and chimeric molecules in the case of TLR997 and TLR9^+/–^ yet still saw dramatic differences. Rather, signal quality was altered, as reflected by numerous transcriptomic differences between signals induced by the parent TLRs and their chimeric counterparts as well as functional outcomes upon in vitro ligand stimulation with CpG DNA.

There are several possible mechanisms by which the TLR9 TIR domain could be inherently less proinflammatory than the TLR7 TIR domain. We provide here some evidence that coupling to MyD88 signaling may be weaker in the context of the TLR9 TIR domain. We recently showed that a TLR9 mutant that cannot bind ligand (*Tlr9^K51E^*) also confers protection from disease, compared with the TLR9 KO (9). We have termed this the “scaffold effect,” as it suggests a regulatory function dependent on protein expression but independent of ligand binding. Further, a TLR9 mutant that can bind ligand but cannot signal via MyD88 (*Tlr9^P915H^*) provides even stronger regulation of disease compared with the TLR9 KO (both of which lack MyD88 signaling), suggests the presence of a MyD88-independent signal-dependent regulatory function (9). Results presented here indicate that regulatory functions of TLR9, which were previously implicated, reside uniquely in the TLR9 TIR domain (9).

Although *Tlr^779^* mice were very protected from disease, a limitation of the work is that TLR779 was only expressed at 50% of WT levels. Because TLR7 dosage has been associated with disease (45–47), this 50% reduction could explain, in part, the suppression of disease. However, on its own, it almost certainly cannot account for the depth of protection observed in *Tlr^779/779^* mice. Indeed, *Tlr^779/779^* mice are as protected from disease as complete *Tlr*7^ko^ mice, even though they express TLR7 at heterozygous levels. Because increases in TLR7 expression lead to increased disease (45–47), one would expect expression at 50% of WT levels to mediate much more disease than a complete knockout. However, that is not what was observed with TLR779, which expresses at 50% of WT levels, yet mediates virtually no more disease than a complete knockout of TLR7. Thus, we conclude that TLR9-TIR plays an active protective role in disease inhibition. Unfortunately, there is no simple technical approach to control the TLR777 expression level to match that of TLR779, since the TLR7 locus is X-linked. Due to X-inactivation that controls the sex dosage compensation process, *Tlr*7^+/–^ females would express 100% of TLR7 dosage in half of their TLR7-expressing cells, while the other half would be TLR7^–/–^, precluding us from using this as a strategy to compensate for the 50% lower expression of TLR^779^.

Critically, the reciprocal phenotype seen when the TLR7 TIR is substituted for the TLR9 TIR in TLR997 does address the differences between the TIRs in the context of DNA-driven stimulation when the amounts of domain-swapped and WT TLR could be fully controlled and matched. Taken together, these reciprocal experiments and complementary results do strongly support the conclusion that TLR9 and TLR7 TIR domains differentially control immunoregulation versus activation. We cannot, however, distinguish whether the ameliorated phenotype of *Tlr^779^* mice is mainly due to the loss of TLR7-TIR activity or to the gain of protective TLR9-TIR.

To determine how TLR9 TIR mediates a different and negative regulatory signal compared with TLR7 TIR will be the next task, which will require new tools and approaches. Though challenging for many reasons, this direction is compelling, as it will open new therapeutic targets to specifically promote TLR9 protective signals and/or inhibit TLR7-TIR in lupus.

In addition to resolving why TLR7 signals drive more severe lupus, our results have implications for fundamental TLR biology. Our work shows that RNA sensing by TLR7 has evolved very differently from DNA sensing by TLR9; DNA sensing is inherently less inflammatory, via linkage of the recognition ectodomain to a less proinflammatory (and in fact, regulatory) signaling TIR domain. The reasons for this are not clear, but may be driven by the durability and ubiquity of extracellular DNA. The constant presence of ligand may have enforced a need to balance the protective effects of pathogen DNA sensing with the risk of autoimmunity. RNA, on the other hand, is very rapidly degraded and, thus, sensing it may be more infrequent and more causally linked to authentic infection.

It is also possible that not all DNA ligands are equal with respect to TLR9 activation. Indeed, 2 ligand binding pockets have recently been identified in the TLR9 ectodomain (48). We can theorize that some ligands might preferentially turn on the proinflammatory or the regulatory pathways. The nature of TLR9 ligands would, in that case, be conditioned by a combination of context-specific ligand and DNAse availabilities (49–52). Hence, it is possible that TLR7 signals can be tuned to a more proinflammatory response than could some TLR9 signals, which, in turn, would benefit the host by enhancing antiviral responses.

Our findings also suggest that important functional differences may exist in the signaling downstream of other TLRs that heretofore have been thought to signal indistinguishably. In a sense, the existence and roles of additional adapters such as TIRAP/MAL and TRIF, which are TLR-specific (e.g., TLR4, TLR2 and TLR3) in their engagement are examples of this (53, 54). However, with respect to other TLRs that are not known to bind different adapters, such possibilities should be further explored.

## Methods

### Sex as a biological variable.

Our study examined male and female animals and similar findings are reported for both sexes.

### Mice.

CRISPR/Cas9 technology was used to generate the strains directly in MRL/lpr mice. The *Tlr^779^* allele was generated in the endogenous *Tlr7* locus; similarly, the *Tlr^997^* allele was generated in the endogenous *Tlr9* locus (Supplemental Figure 5, and Supplemental Methods).

*Tlr9^–/–^* and *Tlr7^–/–^*MRL/lpr mice were previously described (5, 6, 9).

All mice were bred and housed in specific pathogen-free conditions and all experiments were conducted under protocols approved by the University of Pittsburgh IACUC and by the University of Bordeaux.

### Evaluation of clinical disease.

Spleen and axillary lymph nodes were weighed at the time of the takedown. Kidneys were removed, bisected, and formalin fixed. Paraffin embedding, sectioning, and H&E staining were performed by HSRL Stagebio (Mount Jackson, VA). Glomerular and Interstitial nephritis were scored by a pathologist in a blinded manner (Supplemental Methods).

### ELISAs.

Serum antinucleosome total IgG and anti-RNA IgG2a concentrations were determined by ELISA, as previously described (5, 55). The nucleosome-specific antibody clone PL2-3 and the monoclonal antibody BWR4 were used as standards for the antinucleosome and anti-RNA measurements, respectively.

### Flow cytometry.

Spleens were processed via mechanical disruption. Red blood cells were lysed using ammonium-chloride-potassium buffer (prepared in house), and live cells were enumerated with trypan blue and a Luna automated cell counter (Logos Biosystems, Annandale,VA). Five million cells per sample were stained at 100 million cells/mL with the dead cell discriminator Ghost 510 (Tonbo) in PBS (per manufacturers protocol), washed with FACS staining buffer (phosphate buffered saline (PBS) with 3% FBS, 5mM EDTA, and 0.05% sodium azide) and incubated with Fc-R blocking antibody (clone 2.4G2, in house) at 3 g/mL in FACS staining buffer for 15 minutes on ice. To evaluate B cells, suspensions were stained with fluorochrome-conjugated surface antibodies CD19 (clone 1D3, BD), CD45R (clone 6B2, BD), CD23 (clone B3B4, Biolegend), CD21/35 (clone 7E9, Biolegend), CD44 (clone IM7, Biolegend), CD138 (clone 281-2, BD), CD11b (clone M1/70, in house), CD11c (clone N418, in house), CD38 (clone 90, in house), and PNA (clone L-1070, unconjugated antibody Vectorlab, conjugated in our laboratory). T cells were evaluated with antibodies to TCR (clone H57-597, Biolegend), CD4 (clone GK1.5, Biolegend), CD8 (clone 53-6.7, in house), CD62L (clone. MEL-14, Biolegend), and CD44 (clone Pgp-1, in house). Cells were stained for 20 minutes on ice and washed twice with staining media. T cells were fixed with 1% paraformaldehyde (PFA) in PBS, washed and resuspended in staining media. B cells were fixed/permeabilized in a 1% PFA-saponin based Perm/Wash buffer (Cat #554732, BD Biosciences) for 20 minutes, washed twice with 1X BD Perm/Wash buffer and incubated with TLR7 (clone A94B10, BD) or TLR9 (clone J15A7, BD) antibodies in 1X BD Perm/Wash buffer containing 10% mouse serum overnight at 4C. The next day, cells were washed twice with 1X BD Perm/Wash buffer and resuspended in FACS staining buffer. Stained cells were collected using a BD Biosciences LSRII flow cytometer and data were analyzed with FlowJo 10 software. Representative dot plots and gating strategy are shown in Supplemental Figures 6 and 7.

### Analysis of nuclear translocation of NF-κB (p65).

Total splenocytes from 5–7-week-old MRL/lpr mice (of different genotypes as indicated in the figure legends) were isolated as per flow cytometry methods in Stem Cell Buffer (PBS with 2% Fetalplex serum and 1mM EDTA). Cells were warmed at 37C for 45 minutes in media (RPMI 1640 with 10% Fetalplex, Glutamax, Penicillin/Streptomycin, HEPES and 50M of 2-mercaptoethanol) and then stimulated at 10 million cells/ml with CpG DNA (CpG ODN 1826, Hokkaido System Science) and TLR7 agonist CL_097 (Invivogen), as indicated in the figure legends. Cells were fixed with 1.5% PFA for 15 minutes at room temperature and permeabilized in FACS-staining buffer containing 0.1% Triton X-100. Cells were then stained in staining buffer for CD45R (clone RA3-6B2, BD), CD21/35 (clone 7G6, in house), TCR (clone H57-597, Tonbo), and NF-κB-p65 (rabbit polyclonal Santa Cruz Biotechnology) for 45 minutes. Cells were washed and stained with Cy3-conjugated anti-Rabbit secondary antibody (ThermoFisher Scientific) for 20 minutes. Nuclei were stained with DAPI after secondary antibody staining. Data were collected on an Amnis ImageStreamX Mark II Imaging Flow Cytometer and analyzed with the IDEAS software using the “Nuclear localization” feature (EMD Millipore).

### Confocal analysis.

*Tlr^997^-Tlr7^–/–^, Tlr9^+/–^-Tlr7^–/–^* MRL/lpr mice splenocytes (*n* = 2 replicates per genotype, 6–7-week-old female mice) were stained with the live cell discriminator in PBS, washed in stem cell buffer, reconstituted at 200 million cells/ml (2X) with Fc-R blocking antibody (clone 2.4G2, in house) containing stem cell buffer (SCB) and stained with CD19 (clone 1D3, BD) and CD21/CD35 (clone 7E9, in house and Biolegend) for the MZ B cell sort or CD19 (clone 1D3, BD), CD11c (clone N418, in house and ebioscience), and CD11b (clone M1/70, in house) for the ABC sort in SCB, as per cytometry protocol. MZ and ABC B cells were sorted using FACSAria (BD Bioscience) as live singlets CD19^+^ CD21^hi^ and CD19^+^ CD11c^+^ CD11b^+^ cells, respectively. A minimum of 190,000 cells per B cell subset was sorted. Sorted B cells were fixed in a 1.5% PFA-saponin based Perm/Wash buffer (Cat #554732, BD Biosciences) for 15 min at room temperature and for 40 minutes on ice and washed with a 1X Perm/Wash buffer. Cells were stained at 2–10 million cells/ml overnight at 4C in 1X Perm/Wash buffer with fluorochrome-conjugated antibodies CD45R (clone RA3-6B2, Biolegend), TLR7 (clone A94B10, BD), TLR9-biotin (clone Nar-9, from K. Miyake (56), biotin-conjugation in house), and unconjugated EEA1 (polyclonal anti-rabbit, Novus) or LAMP-1 (polyclonal anti-rabbit, Abcam). Cells were washed twice with 1X Perm/Wash buffer and stained with Alexa fluor 488 or 647 conjugated anti-Rabbit secondary antibody (Invitrogen and Jackson Immunoresearch), and/or Alexa fluor 647 conjugated Streptavidin (unlabeled Streptavidin, molecular probes/Thermo Fisher Scientific, conjugated in house) and/or anti-PE-555 conjugated antibody (anti-R-Phycoerythrin, unconjugated, Rockland, Limerick PA, conjugated in house) for 3 hours on ice. Cells were washed twice with 1X Perm/Wash buffer and once with PBS before being mounted on charged slides (Globe Scientific inc, Mahwah, NJ). Data were collected using a Nikon A1 Spectral Confocal and analyzed using NIS Elements and Imaris software (Oxford instruments, Concord, MA) for the 3D reconstruction, using the “spot co-localization” feature. Spots were considered colocalized if the distance between 2 spots was 0.45.

### In vitro RNA-seq collection and analysis.

Total splenocytes were isolated as per flow cytometry protocol in Stem Cell Buffer from *Tlr^779^* *Tlr9^–/–^*, *Tlr7^+/+^ Tlr9^–/–^* 5–7-week-old male MRL/lpr mice and from *Tlr^997^ Tlr7^–/–^*, *Tlr9^–/–^ Tlr7^–/–^* 5-week-old female MRL/lpr mice. Splenocytes were incubated with Fc-R blocking antibody (clone 2.4G2) and rat serum for 5 minutes on ice. Splenic B cells were enriched by negative selection using a biotin conjugated antibody cocktail (CD43 (clone S7, in house), CD4 (clone GK1.5, in house), CD8 (clone TIB105, in house), CD11b (clone M1/70, in house), CD11c (clone N418, in house), Gr-1 (clone RB6.8C5, Biolegend)), followed by magnetic bead-depletion of labeled cells (B cell purity 91%). Bead-purified B cells (4–5 million per conditions) were resuspended in B cell media (RPMI 1640 with 10% Fetalplex, Glutamax, Penicillin/Streptomycin, HEPES and 50M of 2-mercaptoethanol) at 10 x 10^6^ cells/ml, warmed at 37°C for 45 minutes and stimulated for 4 hours with TLR7 agonist CL097 (Invivogen) at 5 μg/ml or TLR9 agonist CpG at 10 μg/ml or left unstimulated as indicated in the figure legends. At the end of the culture, all cells were washed twice with cold PBS. RNA was isolated using the RNeasy Plus Mini Kit (QIAGEN).

### RNA-seq library generation.

RNA was assessed for quality using an Agilent TapeStation 4150/Fragment Analyzer 5300 and RNA concentration was quantified on a Qubit FLEX fluorometer. Libraries were generated with the Illumina TruSeq Stranded mRNA Library Prep kit (Illumina: 20020595) according to the manufacturer’s instructions. Briefly, 100 ng of input RNA was used for each sample. Following adapter ligation, 15 cycles of indexing PCR were completed, IDT for Illumina– TruSeq RNA CD Indexes (Illumina: 20019792). Library quantification and assessment was done using a Qubit FLEX fluorometer and an Agilent TapeStation 4150/Fragment Analyzer 5300. Libraries were normalized and pooled to 4 nM by calculating the concentration based off the fragment size (base pairs) and the concentration (ng/l) of the libraries.

### Library sequencing.

Sequencing was performed on an Illumina NextSeq 500, using a HO150 flow cell. The pooled library was loaded at 4 pM and sequencing was carried out with read lengths of 2x75 bp, with a target of 40 million reads per sample.

Sequences were aligned to the mm10 genome using the STAR aligner (57). Gene-level counts were determined using featureCounts (version v2.0.1) (58) and raw counts were quantile normalized to each other for differential expression using the voom method (59) in the Limma R package (60). For normalization of the datasets, the Quantile method was used. All gene-set enrichments were performed using the rankSumTestWithCorrelation function in limma (version 3.52.2), which explicitly corrects for correlation among genes in the gene set being interrogated.

The Reactome database was utilized to perform pathway enrichment analysis. Heatmap, volcano plot, and bubble plots were built using ggplot2 (version 3.2.1) in R (version 3.6.1).

### In vitro B cell proliferation and differentiation assay.

Spleens were processed via mechanical disruption. Red blood cells were lysed using ammonium-chloride-potassium buffer (prepared in house) and total splenocytes were enumerated with eosin. B cells were magnetic-bead sorted by negative staining with biotin-conjugated antibodies CD43 (Clone S7, BD), CD8a (Clone 53-6.7, Biolegend), CD11b (Clone M1/70, Biolegend), Ly-6G/Ly-6C (Clone RB6-8C5, Biolegend), and CD4 (Clone GK1.5, Biolegend). Cells were first saturated with FcR blocking antibody (clone 2.4G2, BD) and Rat Serum in Stem Cell Buffer (1X PBS, 2% Fetal Bovine Serum, 2mM EDTA) for 5 minutes at 4°C and incubated with antibodies mix for 15 minutes at 4°C, washed with Stem Cell Buffer, incubated with anti-biotin microbeads (#130-090-485, Miltenyi Biotec, Bergisch-Gladbach, Germany) for 5 minutes at 4°C and sorted using LS columns (#130-042-401, Miltenyi Biotec) and QuadroMACS Separators (Miltenyi Biotec). Purity was checked by flow cytometry and was over 95%. Five million B cells were labeled with Violet Proliferation Dye as follows: cells were washed once in cold PBS, and were resuspended in 1 ml of PBS. The dye was then added at a final concentration of 4 mol/ml for 5 minutes at 37°C, quenched and washed twice with B cell media (RPMI 1640 with 10% Fetal Bovine Serum, 1% Glutamine, 1% Penicillin/Streptomycin, HEPES and 50M of 2-mercaptoethanol). B cells were then resuspended in B cell media at 4 x 10^6^/ml and rested for 30 minutes at 37°C. 200,000 cells were plated per well in 96 well plates in B cell media with CpG (ODN 1826, Miltenyi Biotec) at 1 g/mL. Cells were cultured at 37°C for 24, 48, and 72 hours before staining. At each time point, B cells were counted with eosin, stained as described above with Live Dead APC-Cy7 (#65-0865-14, Thermo Fisher Scientific Corporation, Waltham, MA USA), then washed and surface-stained for CD19 (Clone 1D3, BD), CD138 (Clone 281-2, BD) for 15 minutes at 4°C, and washed and intracellularly stained using the FoxP3 Fix/Perm kit (#00-5523-00, Thermo Fisher Scientific Corporation) and antibodies to Blimp-1 (Clone 5E7, Biolegend) and IRF4 (Clone IRF4.3E4, Biolegend) in 1X FoxP3 kit permeabilization buffer overnight at 4°C. The next day, cells were washed twice with 1X FoxP3 kit permeabilization and resuspended in FACS staining buffer. Data were collected using a BD Biosciences LSRFortessa X20 Cell Analyzer. Data were analyzed with FlowJo 10 Software. Representative dot plots and gating strategy are shown in Supplemental Figure 8.

### Statistics.

Statistical significance was quantified with GraphPad Prism software (v.9.5.1) by 2-tailed Mann-Whitney test, 2-tailed unpaired *t* test, multiple paired *t* test, Wilcoxon matched-pairs signed rank test, or 1-way ANOVA, as indicated in each figure. Data distribution was assumed to be normal, but this was not formally tested. A *P* value 0.05 was considered significant.

### Study approval.

All mouse experiments were conducted under protocols approved by the University of Pittsburgh Institutional Animal Care and Use Committee (IACUC) and by the University of Bordeaux.

### Data and materials availability.

RNA-seq data of the in vitro TLR7- or TLR9-stimulated MRL/lpr B cells across *Tlr* genotypes are deposited in the NCBI’s Gene Expression Omnibus database (GEO) under accession numbers GSE202108 and GSE269457. RNA-seq data of the in vitro TLR9-stimulated BALB/c bead-purified B cells, used to generate the TLR9-induced B cell gene signature, are deposited under accession number GSE269458. The mm10 genome database (https://www.ncbi.nlm.nih.gov/assembly/ GCF_000001635.20/) was used to align sequences for the RNA-seq analysis. Values for all data points in graphs are reported in the Supporting Data Values file.

## Author contributions

CL and MJS conceived the project and designed experiments. CL and KBT performed most of the experiments, and CL analyzed the data. CJ performed the B cell activation in vitro experiments and helped with data analysis. RCL helped with confocal experiments. SG created the mice. SB performed the pathologic analysis of the kidney tissue. SJ, DJW, RCL, and KMN assisted with experimental design, data interpretation, and paper edits. CL and SS analyzed and interpreted the RNA-seq data. MJS and CL funded this work. MJS and CL supervised the project. CL and MJS wrote the manuscript.

## Funding support

This work is the result of NIH funding, in whole or in part, and is subject to the NIH Public Access Policy. Through acceptance of this federal funding, the NIH has been given a right to make the work publicly available in PubMed Central.

- ImageStreamX MarkII funded by NIH 1S10OD019942-01 (principal investigator L. Borghesi, Department of Immunology, University of Pittsburgh, Pittsburgh, Pennsylvania, USA).
- NIH grant R37-AI118841.
- French ANR grant TOLLSig ANR-23-CE15-0047 (principal investigator CL).

## Supplementary Material

Supplemental data

Supporting data values

## Version 1 08/12/2025

In-Press Preview

## Version 2 11/03/2025

Electronic publication
