# Supplementary material for: Divergent TIR signaling domains in TLR7 and TLR9 control opposing effects on systemic autoimmunity
Source: J Clin Invest. 2025 Aug 12;135(21):e189566. doi: 10.1172/JCI189566 (PMC12578402; doi:10.1172/JCI189566)
Supplement: Supplemental data [file jci-135-189566-s067.pdf]

Supplemental Material:

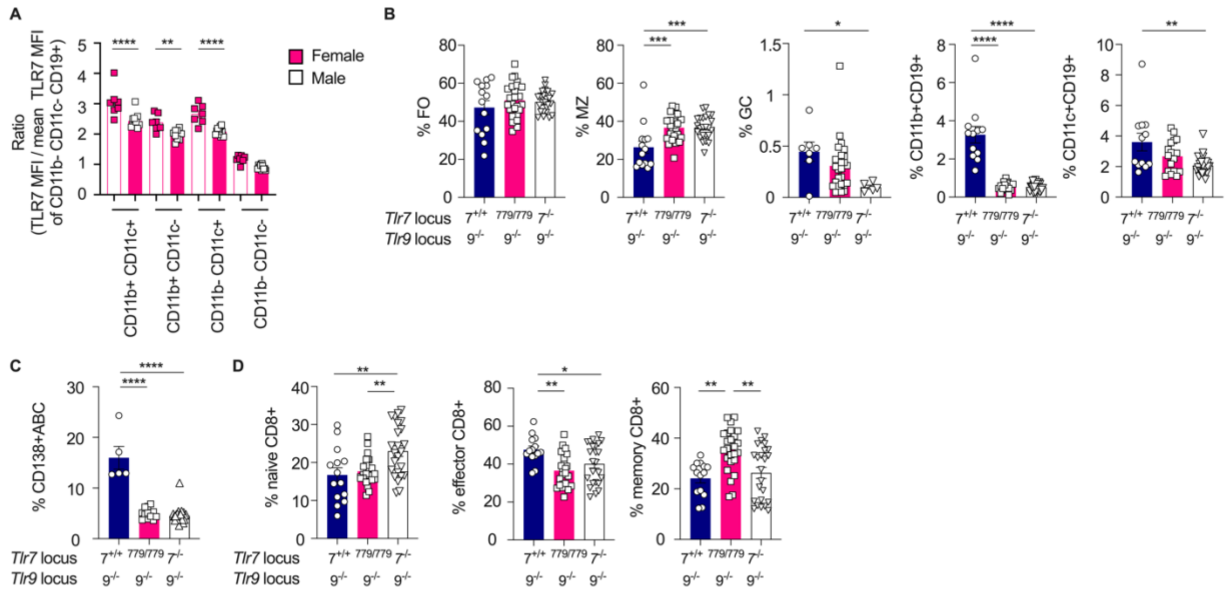

Supplemental Figure 1 (related to Figure 3) Assessment of B and T cell subsets in *Tlr7*$^{+/+}$, *Tlr*$^{779/779}$ or *Tlr7*$^{-/-}$ (all *Tlr9*$^{-/-}$) MRL/lpr mice at disease time points. (A) TLR7 MFI was quantified in different B cell subsets of male (white squares) and female (pink squares) *Tlr*$^{779/779}$ MRL/lpr mice at disease time points. Data are presented as a ratio of TLR7 MFI for individual mice in each subset over the mean TLR7 MFI of the female and male CD11b$^-$CD11c$^-$ subset. Each square corresponds to one individual mouse (female, n=7; male, n=11) **(B)** Percent of CD23^{hi} CD21^{lo} FO, CD23^{lo} CD21^{hi} MZ, CD38^{lo} PNA$^+$ GC, CD11b$^+$ and CD11c$^+$ cells among live B cells (FO, MZ: *Tlr7*$^{+/+}$n=14, *Tlr*$^{779/779}$ n=25, *Tlr7*$^{-/-}$ n=24; GC: *Tlr7*$^{+/+}$n=7, *Tlr*$^{779/779}$ n=25, *Tlr7*$^{-/-}$ n=5; CD11b$^+$, CD11c$^+$: *Tlr7*$^{+/+}$n=12, *Tlr*$^{779/779}$ n=17, *Tlr7*$^{-/-}$ n=24). (**C**) Percent of CD138$^+$ cells among live CD11c$^+$ CD11b$^+$ ABCs (*Tlr7*$^{+/+}$n=5, *Tlr*$^{779/779}$ n=12, *Tlr7*$^{-/-}$ n=24). (**D**) Percent of naive (CD62L^{hi} CD44^{lo}), activated (CD62L^{hi} CD44^{hi}), and memory (CD62L^{lo} CD44^{hi}) T cells among live TCR$^+$ CD8$^+$ splenocytes (*Tlr7*$^{+/+}$n=14, *Tlr*$^{779/779}$ n=25, *Tlr7*$^{-/-}$ n=24). For all panels, data points indicate individual mice and bars indicate the mean +/- s.e.m. The groups are labeled *Tlr7*$^{+/+}$*,* *Tlr*$^{779/779}$ or *Tlr7*$^{-/-}$ if both males and females are included. For statistics,

*P<0.05, **P<0.01, ***P<0.001, ****P<0.0001, one way ANOVA with Tukey's multiple comparisons test.

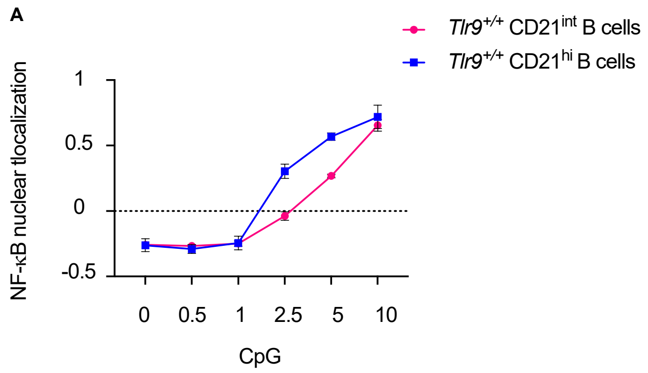

Supplemental Figure 2 (related to Figure 4) Titration of CpG doses

(A) Splenocytes from 8–10-week-old *Tlr9*$^{+/+}$*Tlr7*$^{+/+}$ female BALB/c mice were stimulated with different doses of TLR9 agonist (CpG, µg/ml) for 120 min. Quantification of the NF-κB nuclear localization score in FO CD21^{int} (pink) or MZ CD21^{hi} (blue) B cells. Data points indicate the mean score quantified for n=2 replicates of 3 mice pooled.

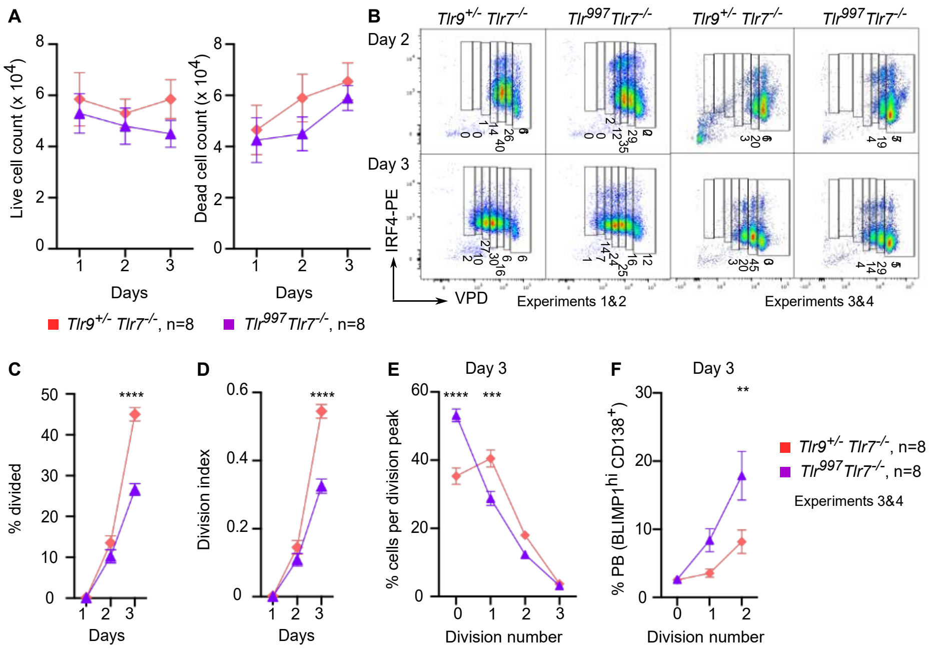

Supplemental Figure 3 (related to Figure 6): TLR997 and TLR999 differentially impact B cell differentiation and proliferation.

Bead purified B cells from 5–7-week-old *Tlr9*$^{+/-}$ or *Tlr*^{997} (all *Tlr7*$^{-/-}$) male and female MRL/lpr mice were labeled with violet proliferation dye (VPD) and cultured for 1, 2 or 3 days with CpG stimulation (1µg/ml), then analyzed by flow cytometry. **(A)** The number of live and dead cells per well were enumerated using eosine stain. Symbols represent means with error bars representing s.e.m from n=8 mice per genotype, cultured individually over four experiments. **(B)** An example of the gating strategy that was used to define the division numbers among live *Tlr9*$^{+/-}$ or *Tlr*^{997} B cells at days 2 and 3. The proliferation profile was different between experiments 1and 2 and experiments 3 and 4. There were 3 and 5 divisions identified at days 2 and 3 respectively for experiments 1 and 2, and 1 and 2 divisions for experiments 3 and 4. This led us to analyze the proliferation data separately (n=2 mice per genotype per experiment). Results from experiments 3 and 4 are presented in C-F **(C)** Percentage of live B cells that divided at least one at the indicated time points post CpG stimulation **(D)** The

FlowJo Proliferation Platform analysis was used to determine the division index. (C-D) Symbols represent means with error bars representing s.e.m from n=4 mice per genotype, cultured individually over two experiments. One-way ANOVA with Sidak's multiple comparisons test was used to compare both genotypes at days 2 and 3. (**E**) Among each division shown in (D) at day 3, the percentage of cells was plotted. Division 0 corresponds to undivided cells. (**F**) Analysis of the effect of TLR999 or TLR997 on B cell differentiation. The percentage of $BLIMP1^{hi}$ $CD138^{+}$ PB (as gated in Figure 6A) for each division number was plotted for both genotypes at day 3. (E-F) Symbols represent means with error bars representing s.e.m from n=4 mice per genotype derived from two experiments. One-way ANOVA with Sidak's multiple comparisons test was used to compare both genotypes at each division number. For all panels * $P < 0.05$, ** $P<0.01$, *** $P<0.001$, **** $P<0.0001$.

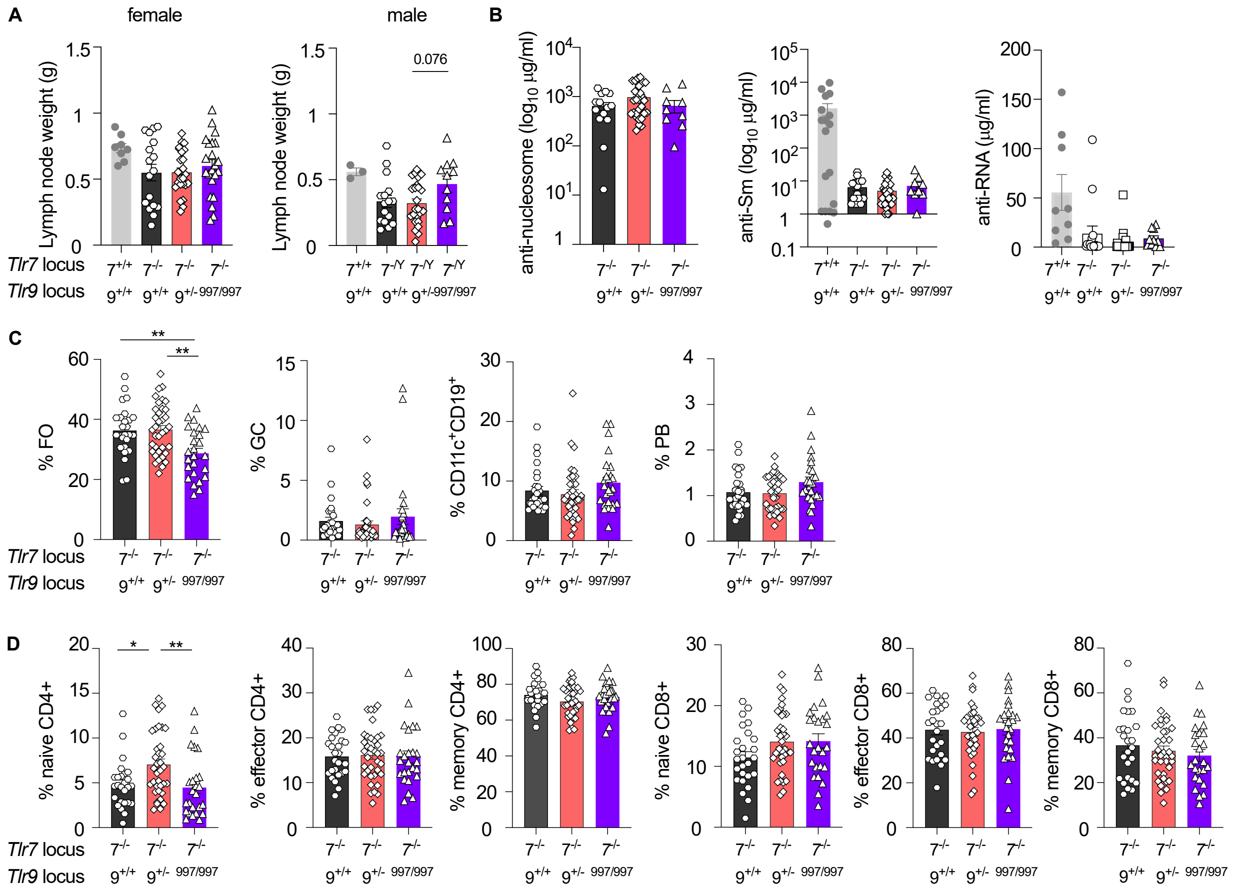

Supplemental Figure 4 (related to Figure 7) Assessment of disease parameters in *Tlr9*$^{+/+}$, *Tlr9*$^{+/-}$ or *Tlr*^{997} (all *Tlr7*$^{-/-}$) MRL/lpr mice at disease time points. 18–21-week-old *Tlr9*$^{+/+}$, *Tlr9*$^{+/-}$ or *Tlr*^{997} (all *Tlr7*$^{-/-}$) MRL/lpr mice were assessed for disease endpoints. Disease endpoints were also assessed in 18–20-week-old wild-type *Tlr7*$^{+/+}$ and *Tlr9*$^{+/+}$ MRL/lpr mice as a reference but were not included in the statistical analysis (A). **(A)** Lymph node weights were measured in mice of the indicated gender and genotypes. (female *Tlr9*$^{+/+}$n=17, *Tlr9*$^{+/-}$ n=28, *Tlr*^{997} n=22, *Tlr9*$^{+/+}$ *Tlr7*$^{+/+}$ n=8; male *Tlr9*$^{+/+}$n=16, *Tlr9*$^{+/-}$ n=24, *Tlr*^{997} n=11, *Tlr9*$^{+/+}$ *Tlr7*$^{+/}$ n=3). **(B)** Quantification of anti-nucleosome, anti-Smith and anti-RNA autoantibodies (*Tlr9*$^{+/+}$n=15, *Tlr9*$^{+/-}$ n=29, *Tlr*^{997} n=10). As a positive comparison for anti Sm and anti RNA production, the anti SM and anti RNA titers of the 16–18-week-old wild-type *Tlr7*$^{+/+}$ and *Tlr9*$^{+/+}$ MRL/lpr mice, that were previously shown in Fig.3 were also added in these panels as a reference. They were not included in the statistical analysis. (anti-RNA: reference group n=9 females;

anti-Smith autoantibodies: reference group n=18 males and females). **(C-D)** Splenic B and T cell subsets were assessed by flow-cytometry in 18-week-old male and 16-week-old female mice of the indicated genotypes. (C) Percent $CD23^{hi}$ $CD21^{lo}$ FO, $CD38^{lo}$ PNA^{+} GC, and $CD11c^{+}$ cells among live B cells, and percent of $TCR^{-}CD44^{hi}CD138^{+}$ plasmablasts among live splenocytes in mice of the indicated genotypes ($Tlr9^{+/+}$n=26, $Tlr9^{+/-}$ n=36, Tlr^{997} n=25). (D) Percent of naive ($CD62L^{hi}$ $CD44^{lo}$), activated ($CD62L^{hi}$ $CD44^{hi}$), and memory ($CD62L^{lo}$ $CD44^{hi}$) T cells among live TCR^{+} $CD4^{+}$ and $CD8^{+}$ splenocytes ($Tlr9^{+/+}$n=26, $Tlr9^{+/-}$ n=36, Tlr^{997} n=25). For all panels, data points indicate individual mice and bars indicate the mean +/- s.e.m. For statistics, *p<0.05, **p<0.01, ***p<0.001, ****p<0.0001, one way ANOVA with Tukey's multiple comparisons test.

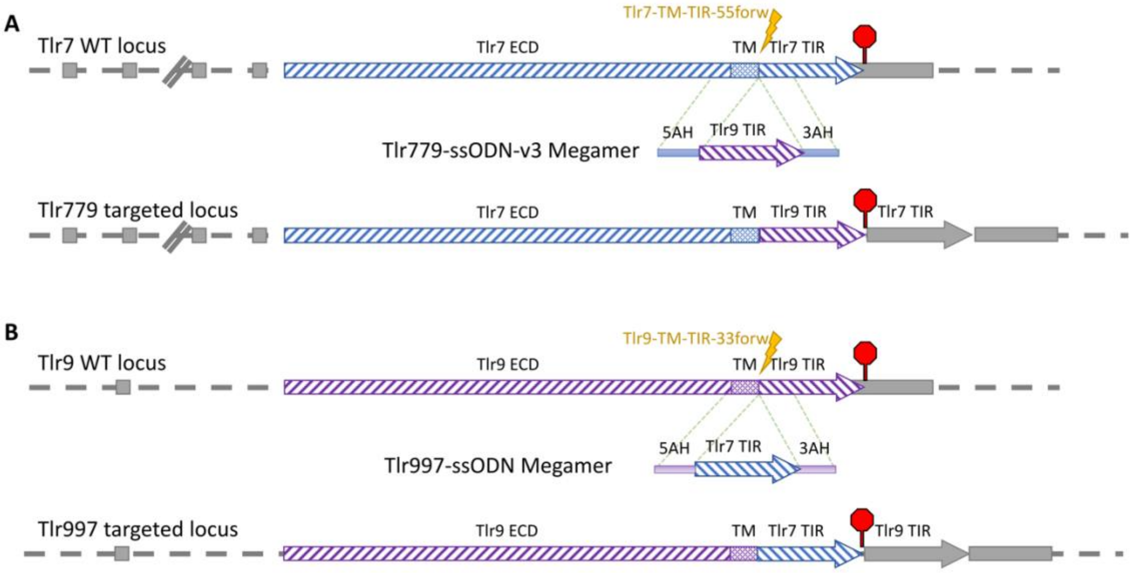

Supplemental Figure 5: Schematic of the targeting strategy to generate the *Tlr*^{779} (A) and *Tlr*^{997} (B) engineered alleles. Genomic sequence features are depicted by box and arrow (*Tlr7* = blue, *Tlr9* = purple). Grey features are non-coding exon or 3'UTR. The SpyCas9 target regions are indicated by a yellow lightning bolt. The dashed lines show the mapping of the homology arms to the germline locus. The transcription stop sites are mark by a red stop sign. ECD: ectodomain. TM: transmembrane domain. TIR: Toll/interleukin-1 receptor/resistance protein domains.

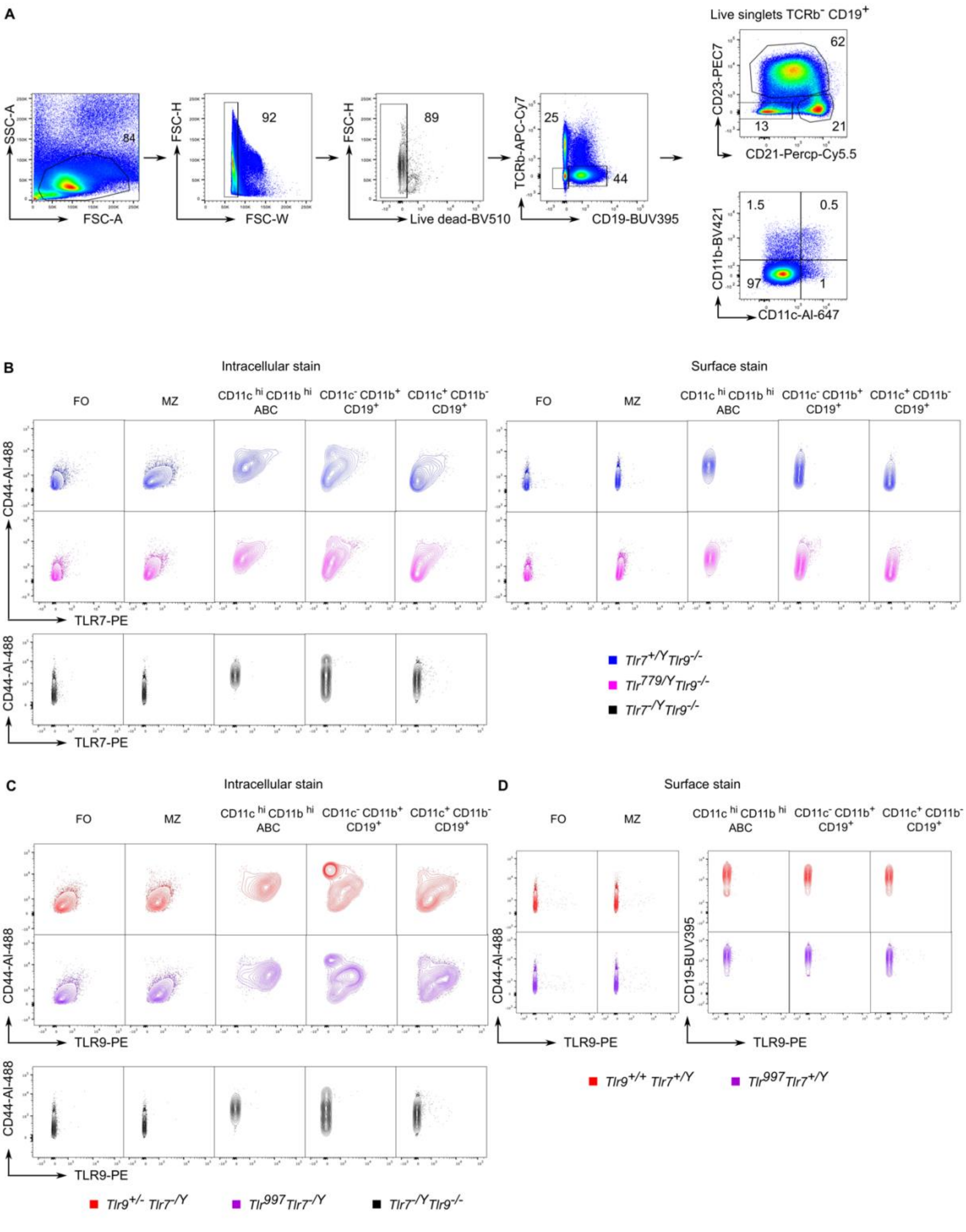

A
SSC-A
FSC-A
84
FSC-H
FSC-W
92
FSC-H
Live dead-BV510
89
TCRb-APC-Cy7
CD19-BUV395
25
44
Live singlets TCRb⁻ CD19⁺
CD23-PEC7
CD21-Percp-Cy5.5
62
13
21
CD11b-BV421
CD11c-Al-647
1.5
0.5
97
1
B
Intracellular stain
Surface stain
FO
MZ
CD11c hi CD11b hi ABC
CD11c⁻ CD11b⁺ CD19⁺
CD11c⁺ CD11b⁻ CD19⁺
CD44-Al-488
TLR7-PE
Tlr7+/Y Tlr9-/-
Tlr779/Y Tlr9-/-
Tlr7-/Y Tlr9-/-
C
Intracellular stain
D
Surface stain
CD44-Al-488
TLR9-PE
CD19-BUV395
Tlr9+/+ Tlr7+/Y
Tlr997 Tlr7+/Y
Tlr9+/- Tlr7-/Y
Tlr997 Tlr7-/Y
Tlr7-/Y Tlr9-/-

Supplemental Figure 6: Representative gating strategy of B cell populations from young MRL/lpr mice.

(A) Representative flow cytometry plots of live, singlet, TCRß$^{-}$CD19+ B cells of 5-7-week-old MRL/lpr mice. Numbers indicate the frequency in the gate. **(B)** Contour plots with outliers showing representative intracellular (left panels) and surface (right panels) staining of TLR7 on live, singlet, TCRß$^{-}$CD19^{+} FO (CD23^{+}CD21^{lo}), MZ (CD23^{lo}CD21^{hi}), CD11c^{+}CD11b^{+}, CD11b^{+} and CD11c^{+} ABCs of 5-week-old *Tlr7*$^{+/Y}$ (blue) or *Tlr*$^{779/Y}$ (pink) (all *Tlr9*$^{-/-}$) male MRL/lpr mice and of 18-week-old *Tlr7*$^{-/Y}$ *Tlr9*$^{-/-}$ (black) male MRL/lpr mice. **(C)** Contour plots with outliers showing representative intracellular staining of TLR9 in Live singlet TCRß$^{-}$CD19^{+} FO (CD23^{+}CD21^{lo}), MZ (CD23^{lo}CD21^{hi}), CD11c^{+}CD11b^{+}, CD11b^{+} and CD11c^{+} ABCs of 5–7-week-old *Tlr9*$^{+/-}$ or *Tlr*^{997} (all *Tlr7*$^{-/-}$) male MRL/lpr mice and of 18-week-old *Tlr7*$^{-/Y}$ *Tlr9*$^{-/-}$ (black) male MRL/lpr mice. **(D)** Contour plots with outliers showing representative surface staining of TLR9 in live, singlet, TCRß$^{-}$CD19^{+} FO (CD23^{+}CD21^{lo}), MZ (CD23^{lo}CD21^{hi}), CD11c^{+}CD11b^{+} CD19^{+}, CD11b^{+} CD19^{+} and CD11c^{+} CD19^{+} of 8-week-old *Tlr9*$^{+/+}$ *Tlr7*$^{+/Y}$ (red) and 13-week-old *Tlr*$^{997/997}$ *Tlr7*$^{+/Y}$ (purple).

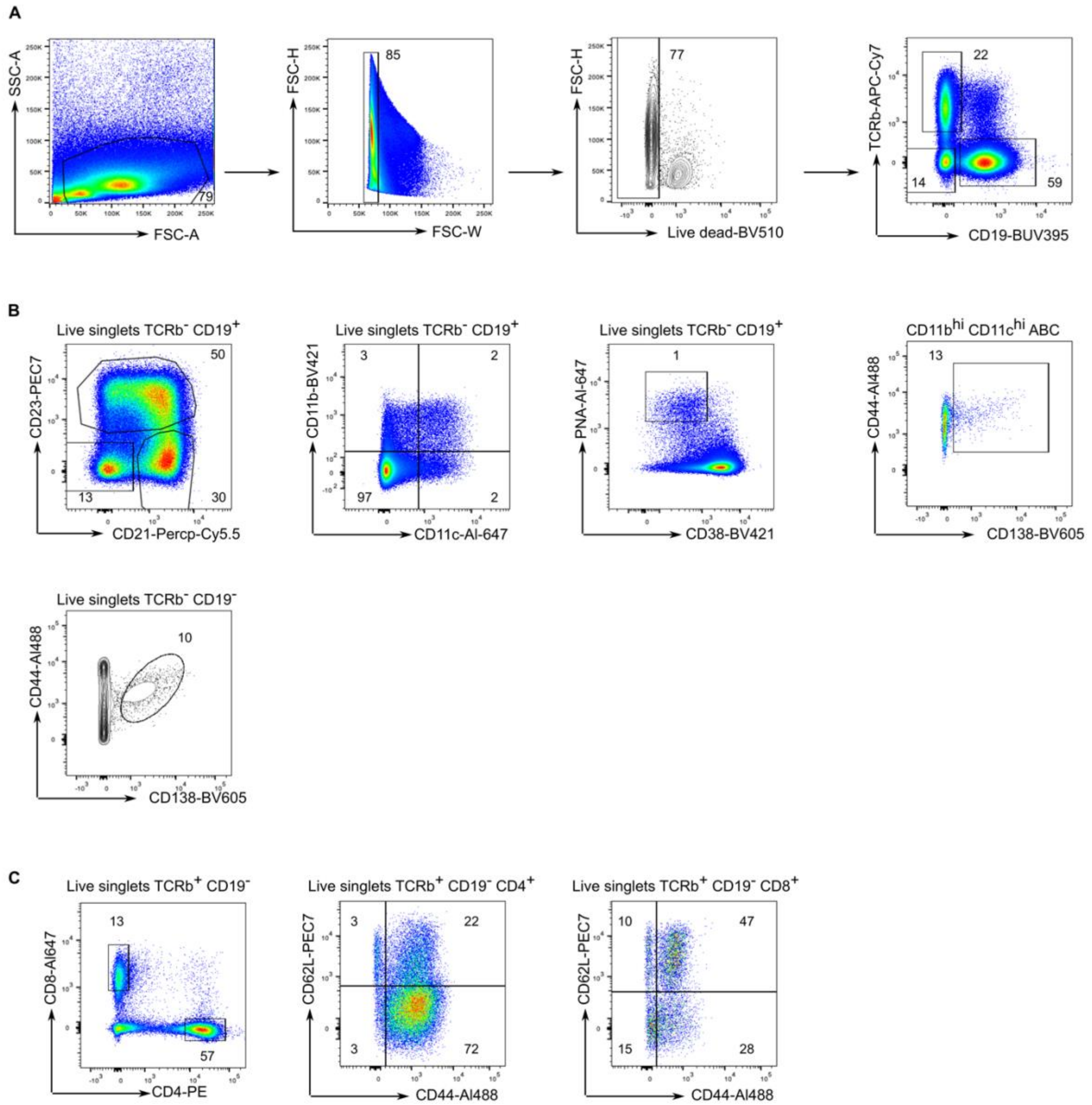
A
SSC-A
FSC-A
79
FSC-H
FSC-W
85
FSC-H
Live dead-BV510
77
TCRb-APC-Cy7
CD19-BUV395
22
14
59
B
Live singlets TCRb⁻ CD19⁺
CD23-PEC7
CD21-Percp-Cy5.5
50
13
30
Live singlets TCRb⁻ CD19⁺
CD11b-BV421
CD11c-Al-647
3
2
97
2
Live singlets TCRb⁻ CD19⁺
PNA-Al-647
CD38-BV421
1
CD11b^hi CD11c^hi ABC
CD44-Al488
CD138-BV605
13
Live singlets TCRb⁻ CD19⁻
CD44-Al488
CD138-BV605
10
C
Live singlets TCRb⁺ CD19⁻
CD8-Al647
CD4-PE
13
57
Live singlets TCRb⁺ CD19⁻ CD4⁺
CD62L-PEC7
CD44-Al488
3
22
3
72
Live singlets TCRb⁺ CD19⁻ CD8⁺
CD62L-PEC7
CD44-Al488
10
47
15
28

Supplemental Figure 7: Representative gating strategy of B and T cell populations from diseased MRL/lpr mice. (A) Representative flow cytometry plots of live, singlet, splenocytes of diseased MRL/lpr mice. **(B)** Representative flow cytometry plots of live, singlet, TCRß$^{-}$CD19^{+} B cell populations. **(C)** Representative flow cytometry plots of live, singlet, TCRß$^{+}$CD19^{-} T cell populations. Numbers indicate the frequency in the gate.

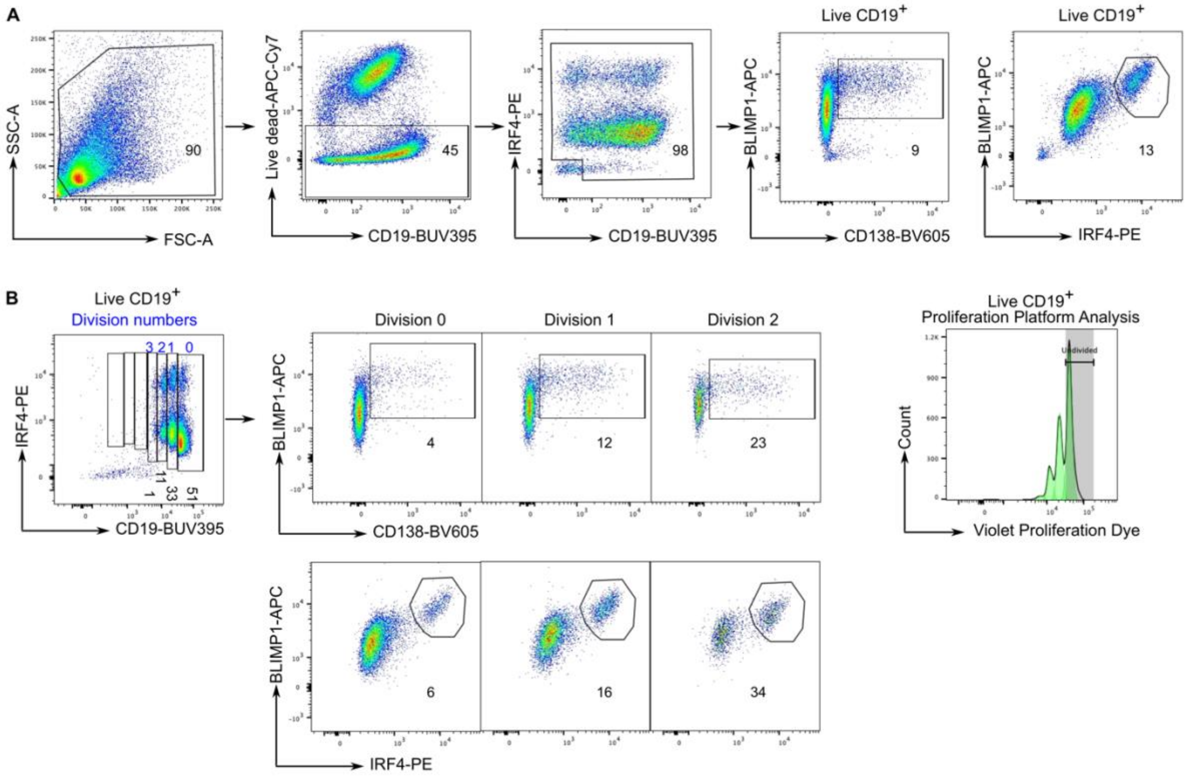

Supplemental Figure 8: Representative gating strategy of the in vitro B cell differentiation from 5–7-week-old MRL/lpr mice. Bead purified *Tlr*^{997} *Tlr7*$^{-/-}$ B cells were cultured for 3 days with CpG (at 1µg/ml), then analyzed by flow cytometry. **(A)** Gating scheme used for analysis of plasmablast (BLIMP1^{+} CD138^{+} and BLIMP1^{hi} IRF4^{hi}) differentiation among live B cells in Figure 6 and Supplemental Figure 2 **(B)** Gating scheme used for analysis of live B cell proliferation in Figure 6 and Supplemental Figure 2. Division numbers are labeled in blue. Division 0 corresponds to undivided cells. The BLIMP1^{+} CD138^{+} and BLIMP1^{hi} IRF4^{hi} gates were applied to all division peaks individually. **(C)** Plot of the FlowJo Proliferation Platform analysis from live B cells.

Supplemental Method

Mice

For each allele, instead of replacing the TIR domain at the endogenous locus, the respective TIR domain, followed by a stop codon and a few bases, was inserted in front of the endogenous TIR. The endogenous TIR sequence was left in place in the genome. This had the advantage of only requiring a single Cas9 target site, which should increase targeting efficiency. Although the endogenous TIR is retained in the genome, now part of the 3'UTR, it cannot be translated because of the stop codon and also because it is out of frame.

The sgRNAs were synthesized by in vitro translation using a PCR-generated template as previously described (1). For the *Tlr*^{779}, the Spy Cas9 target sequence "Tlr7-TM-TIR-55forw" is 5'-ACCTCTTTTTCTGGGATATGTGG-3' (chrX:167,305,882-167,305,904, GRCm38/mm10 Assembly). For *Tlr*^{997} the target sequence "Tlr9-TM-TIR-33forw" 5'-CCTATACTGCACCATCTCTGCGG-3' (chr9:106,226,011-106,226,033, GRCm38/mm10 Assembly).

The targeting strategy was based on the Easi-CRISPR method(2), which used long single stranded oligonucleotides (Megamer, IDT) as a donor template for homology directed repair. The long ssODN sequence correspond to the TIR sequence flanked by about 250 bp for arm of homology derived from the endogenous locus. For the *Tlr*^{779} Megamer, additional silent substitutions were made in the coding sequence of the *Tlr9*-TIR to remove sequence windows with high GC content that would have prevented manufacturing. The sequence of the Tlr7-7-9-ssODN-v3 Megamer was:

TCGCTTTCTTTGCAACTGTGATGCTGTGTGGTTTGTCTGGTGGGTTAACCATACAGATGTTAC
TATTCCATACCTGGCCACTGATGTGACTTGTGTAGGTCCAGGAGCACACAAAGGTCAAAGTG
TCATATCCCTTGATCTGTATACGTGTGAGTTAGATCTCACAAACCTGATTCTGTTCTCAGTTT
CCATATCATCAGTCCTCTTTCTTATGGTAGTTATGACAACAAGTCACCTCTTTTTCTGGGATG
TCTGGTACTGTTTTCATCTGTGCCTGGCATGGCTACCTTTGCTGGCCCGCAGCCGACGCAGCG
CCCAAGCTCTCCCCTATGATGCCTTCGTGGTGTTCGATAAGGCACAGAGCGCAGTTGCGGAC

TGGGTATATAATGAACTCCGCGTCAGGTTGGAAGAGAGGCGAGGGAGAAGAGCCTTGCGAT
TGTGTCTGGAGGACCGAGATTGGCTGCCTGGCCAGACGCTCTTCGAGAACCTCTGGGCTTCC
ATCTATGGGAGCCGCAAGACTCTATTTGTGCTGGCCCACACGGACCGCGTCAGTGGCCTCCT
GCGCACCAGCTTCCTGCTGGCTCAGCAGCGCCTGTTGGAAGACCGCAAGGACGTGGTGGTGT
TGGTGATCCTGCGTCCGGATGCCCATAGATCCCGATACGTGCGCCTTCGACAACGACTGTGT
AGACAGAGTGTTCTCTTCTGGCCCCAACAACCTAACGGTCAAGGTGGCTTCTGGGCCCAGCT
GAGTACAGCCCTGACTAGGGACAACCGCCACTTCTATAACCAGAACTTCTGCCGGGGACCTA
CAGCAGAATAGCATGTGGTACATTTATTATTTTGGAAAGCAAAGATAAAGGGGTATCAGCA
TCTGCAATCCATGGAGTCTTGTTATGATGCTTTTATTGTGTATGACACTAAAAACTCAGCTGT
GACAGAATGGGTTTTGCAGGAGCTGGTGGCAAAATTGGAAGATCCAAGAGAAAAACACTTC
AATTTGTGTCTAGAAGAAAGAGACTGGCTACCAGGACAGCCAGTTCTAGAAAACCTTTCCCA
GAGCATACAGCTC.

The sequence of the Tlr9-9-7-ssODN Megamer was:

AGACGTGAGAAGCAACCCTCTGCACTGTGCCTGTGGGGCAGCCTTCGTAGACTTACTGTTGG
AGGTGCAGACCAAGGTGCCTGGCCTGGCTAATGGTGTGAAGTGTGGCAGCCCCGGCCAGCT
GCAGGGCCGTAGCATCTTCGCACAGGACCTGCGGCTGTGCCTGGATGAGGTCCTCTCTTGGG
ACTGCTTTGGCCTTTCACTCTTGGCTGTGGCCGTGGGCATGGTGGTGCCTATACTGCACCATC
TCTTTTTCTGGGATATGTGGTACATTTATTATTTTGGAAAGCAAAGATAAAGGGGTATCAGC
ATCTGCAATCCATGGAGTCTTGTTATGATGCTTTTATTGTGTATGACACTAAAAACTCAGCTG
TGACAGAATGGGTTTTGCAGGAGCTGGTGGCAAAATTGGAAGATCCAAGAGAAAAACACTT
CAATTTGTGTCTAGAAGAAAGAGACTGGCTACCAGGACAGCCAGTTCTAGAAAACCTTTCCC
AGAGCATACAGCTCAGCAAAAAGACAGTGTTTGTGATGACACAGAAATATGCTAAGACTGA
GAGTTTTAAGATGGCATTTTATTTGTCTCATCAGAGGCTCCTGGATGAAAAAGTGGATGTGA
TTATCTTGATATTCTTGGAAAAGCCTCTTCAGAAGTCTAAGTTTCTTCAGCTCAGGAAGAGAC
TCTGCAGGAGCTCTGTCCTTGAGTGGCCTGCAAATCCACAGGCTCACCCATACTTCTGGCAG

TGCCTGAAAAATGCCCTGACCACAGACAATCATGTGGCTTATAGTCAAATGTTCAAGGAAAC AGTCTAGCTTGCGGCTGGGACGTCTGGTACTGTTTTCATCTGTGCCTGGCATGGCTACCTTTG CTGGCCCGCAGCCGACGCAGCGCCCAAGCTCTCCCCTATGATGCCTTCGTGGTGTTCGATAA GGCACAGAGCGCAGTTGCGGACTGGGTGTATAACGAGCTGCGGGTGCGGCTGGAGGAGCGG CGCGGTCGCCGAGCCCTACGCTTGTGTCTGGAGGACCGAGATTGGCTGCCTGGCCAGACGCT CTTCGAGAA.

MRL/lpr zygotes were produced by in vitro fertilization. The zygotes were injected 0.3 μM EnGen Cas9 protein (New England Biolabs, Cat.No. M0646T), 21 ng/μl CRISPR sgRNA (≈ 0.66 μM) and 10 ng/μl Megamer (IDT). Injected zygotes that developed to the two-cell stage were transferred to the oviducts of pseudopregnant female CD1 recipients to obtain potential founders.

Founder mice were PCR genotyped, using primer pairs that were (i) specific for the integration of TLR9-TIR or TLR7-TIR in *Tlr*^{779} mice and *Tlr*^{997} mice, respectively and (ii) amplified fragments spanning the 5' or the 3' junctions. The targeted alleles of the founders that had correctly integrated were fully sequenced (extending past the junction of the donor DNA and the endogenous locus).

The primer pairs used for genotyping *Tlr*^{779} founders were: primer pair #1 AAGAGCCTGAAGCTGCTGAG (Forward) and GATTGTCTGTGGTCAGGGCA (Reverse), primer pair #2 ACTCAGCTGTGACAGAATGG (Forward) and GGACGCAGGATCACCAACA (Reverse); primer pair #3 GGTTCCAAGGTCTGGTCAAC (Forward) and GATTGTCTGTGGTCAGGGCA (Reverse); primer pair #4 GGTTCCAAGGTCTGGTCAAC (Forward) and TTGCAGGCCACTCAAGGAC (Reverse); primer pair #5 AAGAGCCTGAAGCTGCTGAG (Forward) and TTGCAGGCCACTCAAGGAC (Reverse); primer pair #6 AAGAGCCTGAAGCTGCTGAG (Forward) and GGACGCAGGATCACCAACA (Reverse).

The primer pairs used for genotyping *Tlr*^{997} founders were: primer pair #1 CTCCTTGGCCAAAAATGGGC (Forward) and GGACGCAGGATCACCAACA (Reverse), primer pair #2 CTATGGGAGCCGCAAGACTC (Forward) and GATTGTCTGTGGTCAGGGCA (Reverse).

Subsequent Tlr^{779} progeny were PCR genotyped using the primer pairs #7 CTCCTTGGCCAAAAATGGGC (Forward) and GATTGTCTGTGGTCAGGGCA (Reverse); primer pairs #8 CTCCTTGGCCAAAAATGGGC (Forward) and GGACGCAGGATCACCAACA (Reverse).

Subsequent Tlr^{997} progeny were PCR genotyped using the primer pairs #3 GGCATGGTGGTGCCTATACT (Forward) and GGACGCAGGATCACCAACA (Reverse), and #4 GGAGCTCTGTCCTTGAGTGG (Forward) and AGGTTCTCGAAGAGCGTCTG (Reverse).

Potential off-target deletions of CRISPR-Cas9 strategy were eliminated by backcrossing the founders to MRL/lpr strains (from Jackson Lab or bred locally from Jackson Lab) for at least 3 generations before use in experiments.

MRL/lpr mice were between the ages of 5 to 7 weeks for the analyses of TLR expression and signaling. $Tlr^{779/779}Tlr9^{-/-}$, $Tlr7^{+/+}Tlr9^{-/-}$ and $Tlr7^{-/-}Tlr9^{-/-}$ and $Tlr7^{+/+}Tlr9^{+/+}$ MRL/lpr 16-week-old females and 18-week-old males were assessed for disease. $Tlr^{997}Tlr7^{-/-}$, $Tlr9^{+/-}Tlr7^{-/-}$ and $Tlr9^{+/+}Tlr7^{-/-}$ and $Tlr7^{+/+}Tlr9^{+/+}$ MRL/lpr 18-week-old females and 20-week-old males were assessed for disease. If mice were severely moribund before the experimental endpoint, they were euthanized for humane concerns and included in the analysis. This was the case for n=7 $Tlr7^{+/+}Tlr9^{-/-}$ female mice. Thus, the mean age of $Tlr7^{+/+}Tlr9^{-/-}$ female mice at disease evaluation was 14.78, compared to 16.19, 15.87 and 16.19 for $Tlr7^{+/+}Tlr9^{+/+}$, $Tlr^{779/779}Tlr9^{-/-}$ and $Tlr7^{-/-}Tlr9^{-/-}$ female mice respectively.

1. Pelletier S, Gingras S, and Green DR. Mouse genome engineering via CRISPR-Cas9 for study of immune function. *Immunity.* 2015;42(1):18-27.
2. Miura H, Quadros RM, Gurumurthy CB, and Ohtsuka M. Easi-CRISPR for creating knock-in and conditional knockout mouse models using long ssDNA donors. *Nat Protoc.* 2018;13(1):195-215.

Evaluation of clinical disease

Glomerulonephritis was scored on a scale of 1-6 (1, normal kidney; 2, mesangial expansion and increased mesangial cellularity with patent capillary loops; 3, enlarged glomeruli with moderate endocapillary cellularity; 4, three characteristics with marked endocapillary hypercellularity and loss of patency of most capillary loops; 5, few glomeruli with necrosis (karyorrhexis) or few active (cellular or fibrocellular) or

organized (fibrous) crescents; 6, many active (cellular or fibrocellular) or organized (fibrous) crescents, necrosis (karyorrhexis), obliteration of glomerular architecture, segmental/global sclerosis). Interstitial nephritis was scored on a scale of 1 to 4 in a blinded manner (1, minimal inflammation confined to the perivascular area; 2, expansion of inflammation throughout the interstitial space but maintained in discrete area; 3, diffuse infiltrates in over 40% of high-powered fields; 4, diffuse infiltrate throughout the entire interstitial space).
